# mHealth Social Support Versus Standard Support for Diabetes Management in Safety-Net Emergency Department Patients: Randomized Phase-III Trial

**DOI:** 10.2196/56934

**Published:** 2025-04-23

**Authors:** Elizabeth Burner, Danielle Hazime, Michael Menchine, Wendy Mack, Janisse Mercado, Adriana Aleman, Antonio Hernandez Saenz, Sanjay Arora, Shinyi Wu

**Affiliations:** 1 Department of Emergency Medicine Keck School of Medicine University of Southern California Los Angeles, CA United States; 2 University of Southern California Los Angeles, CA United States; 3 Department of Population and Public Health Sciences Keck School of Medicine University of Southern California Los Angeles, CA United States; 4 Department of Emergency Medicine Los Angeles General Medical Center Los Angeles, CA United States; 5 Suzanne Dworak-Peck School of Social Work University of Southern California Los Angeles, CA United States; 6 Viterbi School of Engineering University of Southern California Los Angeles, CA United States

**Keywords:** social support, mobile health, mHealth, SMS text messaging, diabetes self-management

## Abstract

**Background:**

Mobile health (mHealth) is a low-cost method to improve health for patients with diabetes seeking care in safety-net emergency departments, resulting in improved medication adherence and self-management. Additions of social support to mHealth interventions could further enhance diabetes self-management by increasing the gains and the postintervention maintenance.

**Objective:**

We assessed outcomes of an unblinded, parallel, equal-allocation randomized phase-III trial that tested a social support mHealth intervention to improve emergency department patients’ diabetes self-management.

**Methods:**

Patients with glycated hemoglobin (HbA_1c_) levels of ≥8.5% mg/dL and a text-capable phone were recruited during their emergency department visit for any reason (diabetes related or not) at a US public hospital along with a friend or family member as a supporter. Patients received 6 months of the Trial to Examine Text Messaging in Emergency Department Patients With Diabetes self-management mHealth program. Supporters were randomized to receive either (1) an mHealth social support program (Family and Friends Network Support)—daily SMS text messages guiding supporters to provide diabetes-related social support—or (2) a non-mHealth social support program as an active control—pamphlet-augmented social support with Family and Friends Network Support content. Point-of-care HbA_1c_ level, self-reported diabetes self-care activities, medication adherence, and safety events were collected. Mixed-effects linear regression models analyzed group differences at the end of the intervention (6 months) and the postintervention phase (12 months) for HbA_1c_ level and behavioral outcomes.

**Results:**

A total of 166 patients were randomized. In total, 8.4% (n=14) reported type 1 diabetes, 66.9% (n=111) reported type 2 diabetes, and 24.7% (n=41) did not know their diabetes type; 50% (n=83) reported using insulin for diabetes management. Trial follow-up was completed with 58.4% (n=97) of the patients at 6 months and 63.9% (n=106) of the patients at 12 months. Both groups showed significant HbA_1c_ level improvements (combined group change=1.36%, SD 2.42% mg/dL; 95% CI 0.87-1.83; *P*<.001), with no group difference (group mean difference=0.14%, SD 4.88% mg/dL; 95% CI −1.11 to 0.83; *P*=.87) at 6 months. At 12 months, both groups maintained their improved HbA_1c_ levels, with a combined mean change from 6 months of 0.06% (SD 1.89% mg/dL; 95% CI −0.34 to 0.47; *P*=.76) and no clinically meaningful difference between groups. No differences were observed in safety events. In subgroup analyses, patients recently diagnosed with diabetes in the mHealth social support group improved their glycemic control compared to the standard social support group (between-group difference of 1.96%, SD 9.59% mg/dL; 95% CI −3.81 to −0.125; *P*=.04).

**Conclusions:**

A 6-month change in HbA_1c_ level did not differ by mode of social support in persons using an existing patient-focused mHealth diabetes self-management program, but both groups improved in self-management and glycemic control. Newly diagnosed patients with diabetes benefited most from mHealth-augmented social support.

**Trial Registration:**

ClinicalTrials.gov NCT03178773; https://clinicaltrials.gov/study/NCT03178773

**International Registered Report Identifier (IRRID):**

RR2-10.1016/j.cct.2019.03.003

## Introduction

### Background

Social support interventions using family members and peers to provide emotional and informational support for patients with diabetes have shown improvements in patient motivation, healthy behaviors, and glycemic control [1-3]. However, typical social support interventions require (1) in-person training of family and friends, (2) coordination of schedules and physical location between the patient and their supporter, and (3) the cost of providing physical space and personnel to train these supporters. Because training and support usually occur face-to-face, social supporters are often limited to people who are proximate to the patient and have time available to be trained, rather than being the most influential person in the patient’s life. Mobile health (mHealth) can overcome these transportation and time commitment obstacles and increase the scalability of social support interventions.

mHealth-based social support interventions may increase the efficacy and effectiveness of patient-focused mHealth interventions for diabetes self-management. mHealth for diabetes self-management is effective. Improvements in medication adherence and self-care activities have resulted in glycated hemoglobin (HbA_1c_) level improvements of 0.3% to 0.8% [4]. Despite this, a digital divide persists, with less uptake in populations of a lower socioeconomic status and from minoritized backgrounds [5]. SMS text message–based mHealth strategies have successfully been used among vulnerable patient populations in the United States [6], such as CareMessage [7] in federally qualified health centers in Los Angeles County, California, and Rapid Education/Encouragement and Communications for Health among low-income, primary care clinic patients in Nashville, Tennessee [8]. Outside of primary care settings, the Trial to Examine Text Messaging in Emergency Department Patients With Diabetes (TExT-MED) intervention was tested in safety-net emergency department (ED) patients [9]. The original 6-month, fully automated, SMS text message–based TExT-MED curriculum is based on National Diabetes Education Program [10] messages adapted for SMS text message character limits and emphasizes education and behavior changes. Intervention participants improved their HbA_1c_ levels by 0.4% compared to control participants [9]. Particularly in underresourced populations, patient-focused interventions could be strengthened by adding social support for diabetes modules for loved ones of people with diabetes. In addition, social support may be provided and received differently in different cultural contexts, with family and close friendships taking primacy for patients from a more collectivist cultural background [11]. Using mobile training, a patient can select anyone from their social support network regardless of physical location to be a Family and Friends Network Support (*FANS*) provider. Adding this mobile social support module, TExT-MED+FANS, builds on the success of the original TExT-MED intervention by adding an emotional and highly personal touch to enhance results.

### Objectives

Augmenting mHealth interventions with social support is a growing field of research and has the possibility of creating scalable, effective interventions that can translate into clinical care. Studying these interventions within a safety-net population characterized by inadequate disease control and significant constraints on time and travel can show the potential benefits for those facing barriers to accessing care. The ED provides a unique setting to reach patients and their social supporters during a health crisis, when they may be particularly receptive to adopting behavior changes. In this randomized controlled trial, we tested the effect of 2 approaches to social support to augment an existing patient-focused mHealth intervention. The intervention group received the social support curriculum via mHealth, whereas the control group received the social support curriculum via a paper-based format. The full details of the study design and procedures have been published previously [12] and are available at ClinicalTrials.gov (NCT03178773). This paper presents the trial outcomes.

## Methods

### Study Design

This was an unblinded, randomized, parallel, active controlled trial with a 1:1 allocation ratio. All patient participants received an SMS text message–based mHealth curriculum for diabetes self-management. At the patient level, supporters were randomized to receive (1) the FANS mHealth social support program or (2) the FANS non-mHealth social support program as an active control.

### Ethical Considerations

Institutional review board approval for this study was obtained before study initiation from the University of Southern California Health Sciences Institutional Review Board (HS-17-00406). Patients were consented in the language of their choice (Spanish or English) using written consent documents. Patients repeated back understanding of the study purpose to confirm understanding. Supporters were verbally consented in the language of their choice. All participants had the opportunity to ask questions and obtain clarification on the study purpose. Study data were maintained on HIPAA (Health Insurance Portability and Accountability Act)–compliant servers. The mHealth platform used was HIPAA compliant. Patients were compensated with US $20 at enrollment, US $5 after the 3-month phone survey, US $50 at the 6-month follow-up, US $5 after the 9-month phone survey, and US $100 at the 12-month follow-up. Some patients also came for in-depth semistructured interviews (results reported separately) and received US $100. Supporters were not compensated at enrollment but received US $25 after the 6-month follow-up and US $100 if they came for in-depth semistructured interviews at the end of the study.

### Patient Screening, Eligibility Criteria, and Recruitment

Patients with diabetes were screened and enrolled by surveying the electronic patient tracking system from July 2017 to October 2018. Inclusion criteria were age of ≥18 years, HbA_1c_ level of ≥8.5% mg/dL as measured using the Afinion point-of-care HbA_1c_ analyzer, and ability to consent. Patients were excluded if they did not have stable ownership of a mobile phone for ≥30 days, were not able to send and receive SMS text messages, did not read English or Spanish, or could not identify a support person who could be contacted within 2 weeks to enroll. To identify a support person, patients were asked the following: *Do you have a support person you can count on?* (In Spanish: *¿Tiene una persona de apoyo con la que puede contar?*) Patients who reported type 1, type 2, or unknown type of diabetes were enrolled as prior work with this population has shown that up to 30% of patients are unsure of which type of diabetes they have [13]. Research assistants explained the purpose of the study and obtained consent while the patient was still in the ED. Patients were informed at enrollment that the designated supporter could receive multiple SMS text messages per day and would be prompted to offer increased support.

### Patient and Supporter Enrollment and Randomization

After consenting, patients were registered on the mHealth platform but were only eligible to complete study enrollment if a supporter agreed to participate as well. If a supporter was not enrolled within 2 weeks of patient consent, the patient still received the patient SMS text messaging program but was excluded from further participation in the study. Randomization to pamphlet- or mHealth-augmented social support took place after supporter enrollment.

Supporters were enrolled during the initial in-person enrollment of the patient if available in the ED, by telephone, or at a later time remotely. Support person enrollment consisted of verbal consent and confirmation of age of >18 years and ability to send and receive SMS text messages. Randomization group was assigned through sealed envelope allocation after supporter consent to participate; the randomization sequence was generated by the senior study biostatistician. Supporters then completed baseline survey instruments and were registered on the mHealth platform if randomized to the mHealth social support intervention arm or received a pamphlet if randomized to the active control standard support arm.

### Intervention

#### TExT-MED: Patient Intervention (Received by All Patients)

The original 6-month, fully automated, SMS text message–based TExT-MED patient curriculum has been previously described [9]. TExT-MED was based on the National Diabetes Education Program [10] adapted for character limits (160 characters) and emphasized education and behavior changes. The program was designed to enhance knowledge, self-efficacy, and diabetes self-management. Patient messages, delivered twice daily, included (1) educational and motivational messages, (2) medication reminders, (3) trivia questions, and (4) healthy living challenges.

After the original TExT-MED study, Agile Health purchased, modified, and commercialized the program as MyAgileLife. This enhanced version delivered 3 daily messages with a greater focus on skills such as setting goals, enabling social support, and increasing engagement. To synchronize patient and supporter messages, we used a locally modified MyAgileLife version. All patients received the TExT-MED patient intervention.

#### FANS Curriculum: Supporter Intervention

##### Overview

The development of the FANS support curriculum has been described separately [12]. In brief, the messages were developed based on National Diabetes Education Program and American Diabetes Association recommendations, synchronizing in content and time with 2 of the 3 daily patient messages. The FANS messages focused on (1) instrumental support (tangible goods and actions), (2) informational support (knowledge), and (3) emotional support [14]. Given the financial constraints of the patients and family members of this population, FANS messages emphasized nonfinancial forms of instrumental support. One FANS message per week was an active support challenge message that encouraged patient contact, emphasized a specific support care behavior, or challenged the FANS supporters to perform the same health behavior as the patient and communicate that to the patient. In total, the FANS curriculum consisted of 381 messages. All supporters received the FANS curriculum but were randomized to the treatment or active control group—FANS curriculum delivered via mHealth versus non-mHealth methods, respectively.

##### Treatment Condition: Supporters Randomized to FANS Curriculum via mHealth

Supporters in the intervention group received 2 to 3 SMS text messages daily, synchronized in content and time with the patient TExT-MED messages. Messages to supporters started on the same day as the patients’ TExT-MED messages. Research assistants did not provide further guidance to supporters other than to read the messages and an information line to text or call if they had technical difficulties.

##### Active Control Condition: Supporters Randomized to FANS Curriculum via Non-mHealth Methods

Supporters randomized to the active control group received the FANS curriculum delivered through non-mHealth methods. Supporters received a paper pamphlet of the FANS curriculum, with each 2-page layout of the pamphlet corresponding to 1 week of TExT-MED patient messages. The pamphlet was provided directly by a research assistant if the supporter enrolled in person or mailed to the supporter’s home if they enrolled remotely. Each supporter was instructed to start on week 1 of the pamphlet on the same day that their patient’s messages would start (the dyad’s *Healthy Start Date*). Research assistants did not provide further guidance to supporters on how to text or provide social support. An information telephone number was provided in case they had technical difficulties.

### Safety Monitoring

There was a potential risk of hypoglycemia as patients improved their medication adherence or physical activity, especially if patients were previously prescribed insulin or oral insulin secretagogues at increasing doses due to persistent hyperglycemia. Patient knowledge of symptoms of and treatment for hypoglycemia is low [13]; thus, hypoglycemia was the first focus of the educational messages sent to both supporters and patients. Upon enrollment, patients were instructed to report episodes of hypoglycemia to the research team and call their primary care team. If the patient did not have a regular primary care source, the research team instructed the patient to visit the urgent access center at the medical center where they were initially enrolled. We evaluated for a difference in patient-reported hypoglycemic events between the 2 groups at 6 and 12 months.

### Data Collection Procedures, Schedule, and Outcome Measures for Patients

Patient assessments took place at enrollment and 3, 6, 9, and 12 months for behavioral and psychosocial outcomes and at baseline and 6 and 12 months for clinical outcomes. Trained research assistants conducted in-person assessments at an office at the medical center using standardized protocols and equipment in the patient’s preferred language. For assessments at 3 and 9 months, participants had the option of an in-person, mail, or phone appointment. Data were entered into a data management system maintained by the Southern California Clinical and Translational Science Institute [15].

At baseline, we collected self-reports of race and ethnicity, language preference, health literacy (3-item Brief Health Literacy Screen [16]), and mobile technology use measured using questions modeled after the Pew Hispanic Center survey [17].

At baseline and 6 and 12 months, we collected HbA_1c_ using the Afinion AS100 capillary point-of-care machine, systolic blood pressure, weight, and abdominal circumference. We collected patient height only at baseline to calculate BMI.

Patient measures collected at baseline and 3, 6, 9, and 12 months were as follows: (1) the Summary of Diabetes Self-Care Activities [18] (each measure ranges from 0 to 7, indicating the number of days per week that the patient reports engaging in these behaviors), (2) the 3-item medication adherence scale by Wilson et al [19] (total score ranges from 0 to 100, with higher scores indicating better medication adherence), (3) self-efficacy (Diabetes Empowerment Scale–Short Form [20]; ranges from 8 to 40 points; a higher score indicates higher self-efficacy), (4) the Diabetes Distress Scale [21] (an average of 17 Likert-scale items; overall scores range from 1 to 6, with higher scores indicating higher levels of distress), (5) depression (Patient Health Questionnaire–9 [22]; a widely used scale for depression; higher levels indicate more depression symptoms), (6) the Diabetes Fatalism Scale [23] (sum of 3 subscales: emotional distress, religiosity, and coping and perceived self-efficacy; the total score ranges from 12 to 72, with higher scores indicating higher fatalism), (7) the World Health Organization–Five Well-Being Index [24] (a widely used measure of quality life validated in many languages that consists of only 5 items), (8) the Diabetes Family Behavior Checklist supportive and nonsupportive subscores [25] (the supportive subscore ranges from 4 to 45 [higher scores indicate more supportive behaviors]; the nonsupportive subscore ranges from 7 to 35 [higher scores indicate more nonsupportive behaviors]), (9) the Diabetes Care Profile Support Questions [26] (with subscores for support wanted, support received, and support attitudes; each subscore ranges from 5 to 30, with higher scores indicating higher desire for support and higher support received), and (10) the Norbeck Social Support Questionnaire Emotional and Tangible subscales [27] (the emotional subscore ranges from 0 to 16, with higher scores indicating higher perceived emotional support, and the tangible subscore ranges from 0 to 8, with higher scores indicating higher perceived tangible support).

Patient self-reported frequency of patient-supporter contact and proportion of communication about diabetes was also collected.

### Data Collection Procedures, Schedule, and Outcome Measures for Supporters

Supporter assessments took place at baseline and 6 and 12 months. Supporters had the option of an in-person, mail, or phone appointment. All assessments were with trained research assistants in the language of the participants’ preference.

Self-report of age, race and ethnicity, language preference, health literacy [16], and mobile technology use [17] were collected at baseline. At baseline and 6 months, support people reported (1) frequency of patient-supporter contact and the proportion of the communication that was about diabetes and (2) supporter diabetes-related distress (Partner Distress Scale [28]).

### Primary and Secondary Outcomes

The primary outcome was change in HbA_1c_ level from baseline to 6 months. The secondary outcomes were (1) 6- to 12-month postintervention change in HbA_1c_ level, (2) baseline to 6-month change in BMI and blood pressure, and (3) baseline to 6-month change in diabetes self-management behaviors (measured using the Summary of Diabetes Self-Care Activities)

### Data Analysis

#### Primary Outcome Analysis

The primary outcome was the change in HbA_1c_ level from baseline to 6 months between the mHealth social support and standard social support groups. Participants who completed the 12-month study provided 2 outcome measures of 6-month change: a 0- to 6-month measure of treatment efficacy and a 6- to 12-month measure of sustainability of treatment effect. The normality of the outcome variable (6-month change) was graphically evaluated. We used a mixed-effects linear regression model to account for correlated outcome data (0-6–month and 6-12–month changes) and loss to follow-up. Analyses were conducted using intention to treat, with participants analyzed according to their randomized intervention regardless of adherence. The linear mixed-effects model included a random intercept term for participants. Fixed effects included treatment allocation, initial level of HbA_1c_ (0-month measure for treatment efficacy and 6-month measure for sustainability), and a covariate of study period (0-6 months and 6-12 months). We tested for group differences in the main effect of treatment over both periods of 0 to 6 months and 6 to 12 months. An interaction term of treatment by study period tested for differences in treatment effects by study period; treatment effects were estimated and tested for differences by study period in this interaction model. Model assumptions, including normality of model residuals and homogeneity of variance, were evaluated. All analyses were conducted in Stata (version 17; StataCorp) [29].

Due to the possibility of unequal groups, we planned to examine for potential confounding from candidate variables by the change in intervention efficacy estimation method, with a cutoff of 20% change [12]. Potential confounders examined were sex of the patient, sex of the supporter, patient age, race and ethnic group, language preference, health literacy, patient and supporter technological capacity, patient and supporter living at the same address, baseline family supportive behaviors, and supporter being immediately present for enrollment versus requiring multiple contact attempts.

After the regression model was built, predicted mean differences between the groups in HbA_1c_ levels at 6 months were examined with the margins and contrast postestimation tools in Stata at the 6-month time point. A sensitivity analysis confined to adherent participants (those who had not opted out of messages and had received ≥75% of messages confirmed by the message delivery platform) was planned but was not possible due to limitations in most patients’ cellular service provider platforms.

#### Secondary Outcome Analysis

Secondary outcomes of the 6- to 12-month intervention efficacy of mHealth social support versus standard social support on HbA_1c_ and 0- to 6-month intervention efficacy of mHealth social support versus standard social support on clinical outcomes and self-care behaviors were examined using the same mixed-effects models as for the primary outcome.

#### A Priori Subgroup Analysis

To determine whether subgroups of participants were differentially affected by the intervention, secondary analyses evaluating intervention moderators were planned a priori [12]. For HbA_1c_ and each of the secondary outcomes, interaction terms (randomized intervention-by-moderator product terms) were added to mixed-effects linear models similar to those described previously. Variables evaluated as moderators included patient and supporter sex, race and ethnicity in 4 categories, language preference, health literacy (low health literacy was defined as a Brief Health Literacy Score of <12), new diagnosis of diabetes (<12 months), baseline frequency of mobile technology use as high or low based on latent profiles previously described [30], and physical proximity to supporter. Intervention effects were estimated by levels of the moderator for significant moderators only at a *P* value of .05 for the interaction term between randomization group and moderator.

#### Post Hoc Subgroup Analysis

During enrollment, substantial differences in baseline support and contact between patients and their selected supporters became evident as some supporters took up to 2 weeks to complete enrollment procedures. We conducted a subgroup analysis based on supporter immediate availability for enrollment versus delayed enrollment.

#### Sample Size and Effect Size Calculations

We planned to enroll 166 patient-supporter dyads assuming a 30% loss to follow-up [31] to yield a planned sample size of 116 total dyads. Power of 0.80 and 2-sided α of .05 using an SD of the final HbA_1c_ level of 1.6 (the value of our previous trials) provided the ability to detect a mean difference in change in HbA_1c_ level of 0.84 between the 2 groups at the 6-month follow-up [32].

## Results

### Screening and Recruitment

**Figure 1:**
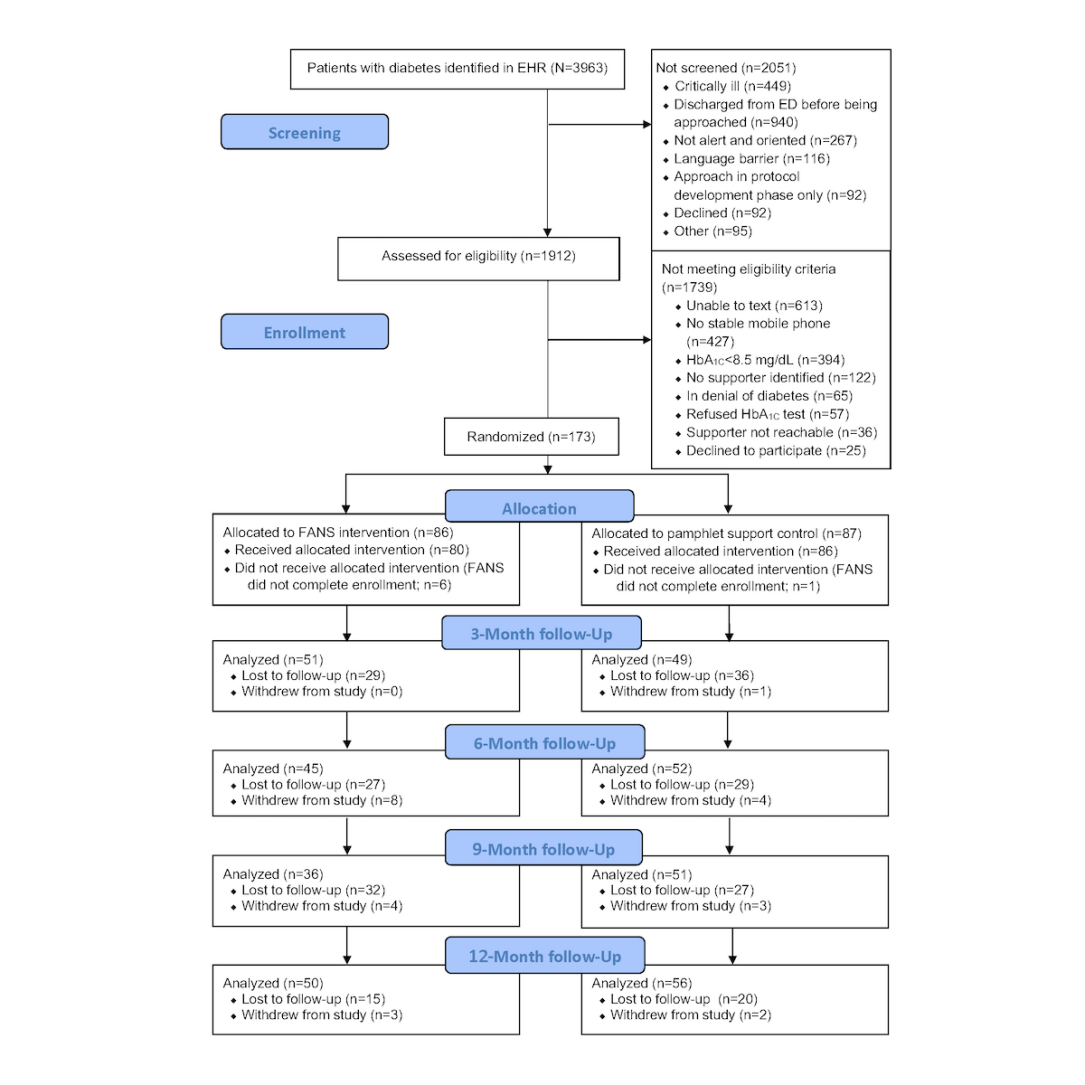
Participant flow diagram. ED: emergency department; EHR: electronic health record; FANS: Family and Friends Network Support; HbA_1c_: glycated hemoglobin.

Nearly 4000 patients with diabetes were identified during their ED visits in the electronic patient tracking system. Nearly half of these patients (1912) were screened for eligibility (see the CONSORT [Consolidated Standards of Reporting Trials] diagram in Figure 1). Of these 1912 patients, 209 (10.93%) were initially recruited, and 173 (8.63%) met the criteria and agreed to enroll. A total of 166 patients had a consenting support person identified and were randomized. The most common reason for ineligibility was not using SMS text messaging (613/1912, 32.06% of patients), followed by not having a stable mobile phone number (427/1912, 22.33% of patients). Less than 10% of patients (156/1912, 8/15%) were unable to identify an available supporter. After randomization, 4% (7/173) of the supporters failed to complete the initial process of enrollment in the study and were excluded. Recruitment ended once the final cohort of 166 patient-supporter dyads was randomized and fully enrolled in the intervention.

### Participant Characteristics

The characteristics and baseline measurements of the patient cohort are shown in Table 1. The enrolled patient cohort was 51.2% (85/166) female, 69.9% (116/166) Spanish speaking, and 77.7% (129/166) born outside the United States, with a median age of 48.2 (IQR 40.7-55) years. Two-thirds (111/166, 66.9%) reported type 2 diabetes, and 50% (83/166) used insulin. Their mean HbA_1c_ level was 10.8 (SD 1.7) mg/dL. Comorbidities were common. Mean systolic blood pressure was 134.6 (SD 24.6) mm Hg, mean BMI was 30.07 (SD 7.60) kg/m^2^, and mean Patient Health Questionnaire–9 score was 9.16 (SD 6.63; mild depression). Medication adherence and diabetes self-care behaviors were low, with mean days of performing recommended daily diabetes self-care activities of 2.47 (SD 2.57) to 4.06 (SD 2.93) and a mean Wilson medication adherence score of 66.5 (SD 29.5). The mHealth social support intervention group consisted of more male individuals (45/80, 56% vs 37/86, 43%) and fewer Spanish speakers (52/80, 65% vs 64/86, 74%) than the standard support control group.

**Table 1:** Patient baseline characteristics (N=166).

			Total		Intervention (n=80)		Active control (n=86)
HbA_1c_^a^ level (%; mg/dL)^b^, mean (SD)			10.84 (1.69)		10.90 (1.73)		10.78 (1.67)
Reason for emergency visit was diabetes related, n (%)			128 (77.1)		60 (75)		68 (79.1)
Insulin-dependent diabetes, n (%)			83 (50)		38 (47.5)		45 (52.3)
BMI (kg/m^2^)^b^, mean (SD)			30.07 (7.60)		29.34 (7.18)		30.78 (7.96)
Age (y), mean (SD)			47.60 (10.43)		46.91 (9.98)		48.25 (10.85)
Systolic BP^c^ (mm Hg)^b^, mean (SD)			134.6 (24.6)		133.8 (25.9)		135.4 (25.8)
Male sex, n (%)			81 (48.8)		45 (56.3)		37 (43)
**Race and ethnicity, n (%)**							
	American Indian or Alaska Native	5 (3)		3 (3.8)		2 (2.3)	
	Asian or Pacific Islander	2 (1.2)		0 (0)		2 (2.3)	
	Black	9 (5.4)		6 (7.5)		3 (3.5)	
	Latino	147 (88.6)		68 (85)		79 (91.9)	
	Non-Hispanic White	3 (1.8)		3 (3.8)		0 (0)	
Spanish language preferred, n (%)			116 (69.9)		52 (65)		64 (74.4)
Foreign born (n=165), n (%)			129 (77.7)		56 (70)		74 (86)
Acculturation (Short Acculturation Scale for Hispanics), mean (SD)			2.01 (1.18)		2.10 (1.21)		1.92 (1.16)
Health literacy (Brief Health Literacy Screen)^d^, mean (SD)			4.60 (3.43)		4.75 (3.54)		4.45 (3.34)
Depression (PHQ-9^e^)^b^, mean (SD)			9.16 (6.63)		9.44 (6.31)		8.90 (6.94)
Self-efficacy (Diabetes Empowerment Scale–Short Form)^b^, mean (SD)			3.85 (0.66)		3.89 (0.62)		3.81 (0.69)
Distress due to DM^f^ (Diabetes Distress Scale)^b^, mean (SD)			2.48 (1.03)		2.63 (1.11)		2.34 (0.93)
Quality of life (WHO-5^g^)^d^, mean (SD)			60.9 (28.00)		58.0 (29.45)		61.9 (26.59)
Fatalism (Diabetes Fatalism Scale)^b^, mean (SD)			34.94 (9.89)		35.86 (9.85)		34.09 (9.90)
Medication adherence (n=165; Wilson 3-item medication adherence scale)^d^, mean (SD)			66.5 (29.5)		65.8 (28.2)		67.29 (30.9)
**Summary of Diabetes Self-Care Activities (d), mean (SD)**							
	General diet^d^	3.23 (2.47)		3.07 (2.51)		3.38 (2.44)	
	Specific diet^d^	3.87 (1.90)		3.74 (1.89)		3.98 (1.90)	
	Glucose monitoring^d^	2.65 (2.92)		2.76 (3.06)		2.55 (2.79)	
	Foot care^d^	4.06 (2.93)		4.11 (2.86)		4.01 (3.00)	
	Carbohydrate spacing^d^	2.89 (2.56)		3.00 (2.46)		2.78 (2.65)	
	Exercise^d^	2.47 (2.57)		2.43 (2.53)		2.51 (2.62)	
**Support measures, mean (SD)**							
	Supportive diabetes family behaviors^d,h^	23.90 (8.87)		24.46 (8.99)		23.37 (8.78)	
	Nonsupportive diabetes family behaviors^b,h^	18.23 (6.61)		18.70 (6.69)		17.78 (6.54)	
	Diabetes support needs^b,i^	23.61 (7.52)		24.04 (7.55)		23.22 (7.51)	
	Diabetes support received^d,i^	18.54 (8.90)		19.16 (9.06)		17.98 (8.77)	
	Diabetes support attitudes^d,i^	6.45 (5.17)		6.39 (4.68)		6.51 (5.62)	
	General emotional support (n=152)^d,j^	13.84 (3.23)		13.7 (3.15)		14.0 (3.32)	
	General tangible support (n=152)^d,j^	6.99 (1.68)		7.15 (1.57)		6.83 (1.77)	

^a^HbA_1c_: glycated hemoglobin.

^b^Higher value indicates clinically worse value.

^c^BP: blood pressure.

^d^Lower value indicates clinically worse value.

^e^PHQ-9: Patient Health Questionnaire–9.

^f^DM: diabetes mellitus.

^g^WHO-5: World Health Organization–Five Well-Being Index.

^h^Diabetes Family Behavior Checklist.

^i^Diabetes Care Profile.

^j^Norbeck Social Support Questionnaire.

### Supporter Characteristics and Baseline Support

The supporters were 70.5% (117/166) female and 57.2% (95/166) Spanish speaking, with 65.7% (109/166) of supporters being born outside the United States (Table 2). Their mean age was 43.69 (SD 14.54) years. A total of 27.7% (46/166) of patient-supporter dyads were language discordant in the language they preferred to receive SMS text messages. Of the supporters, 20.5% (34/166) also had diabetes, 68% (23/34) with type 2 diabetes, 6% (2/34) with type 1 diabetes, and 24% (8/34) who did not know the type of diabetes they had. The supporters were predominantly family members—30.7% (51/166) were spouses, 14.5% (24/166) were siblings, 23.5% (39/166) were adult children of the patients, 16.9% (28/166) were other relatives, 12% (20/166) were friends, and 2.4% (4/166) of the patients did not wish to disclose the nature of their relationship with their supporter.

**Table 2:** Supporter baseline characteristics (N=166).

	Total	Intervention (n=80)	Active control (n=86)
Supporter has diabetes, n (%)	34 (20.5)	16 (20)	18 (20.9)
Age (y), mean (SD)	43.69 (14.54)	42.89 (13.79)	44.45 (15.26)
Male sex, n (%)	49 (29.5)	21 (26.3)	28 (32.6)
Spanish speaking, n (%)	95 (57.2)	48 (60)	46 (53.5)
Foreign born, n (%)	109 (65.7)	52 (65)	57 (66.3)
Supporter enrollment delayed (not the same day), n (%)	67 (40.4)	34 (42.5)	33 (38.4)

### Study Follow-Up

We obtained measures of our primary outcome, HbA_1c_ level at 6 months, from 52% (42/80) of the mHealth support intervention group and 60% (52/86) of the active control standard support group. In the intervention group, 10% (8/80) of the patients dropped out, and 34% (27/80) were lost to follow-up. In the active control group, 6% (5/86) of the patients dropped out, and 34% (29/86) were lost to follow-up. At 12 months, after a maintenance phase with no SMS text messages, we obtained HbA_1c_ measurements for 62% (50/80) of the patients in the intervention group and 65% (56/86) of the patients in the active control group. In the intervention group, 9% (7/80) of the patients withdrew during the maintenance phase, but 6% (5/80) of the patients followed up who were not available at 6 months. In the active control group, 6% (5/86) of the patients withdrew during the maintenance phase, but 5% (4/86) more followed up who were not available at 6 months.

Comparison of the patients who completed or did not complete follow-up at 6 months showed that patients who did not complete the 6-month assessments reported more negative attitudes toward their baseline social support (Diabetes Care Profile support attitudes negative subscore=2.25, 95% CI 1.95-2.55 vs 1.56, 95% CI 1.36-1.76; group difference=−0.69, 95% CI −1.03 to −0.35). Patients unavailable for follow-up were substantially younger (mean age 45.78 y, SD 10.79; 95% CI 43.13-48.43 vs 48.80 y, SD 10.06; 95% CI 46.80-50.80) and more acculturated (Short Acculturation Scale for Hispanics=mean 2.17, SD 1.27; 95% CI 1.86-2.49 vs mean 1.90, SD 1.11; 95% CI 1.68-2.12), although these differences were not significantly different (Multimedia Appendix 1).

### Primary Outcome: 6-Month Change in HbA_1c_ Level

The mHealth support intervention and non-mHealth standard support active control condition were similarly efficacious in improving glycemic control (HbA_1c_), with no clinically meaningful difference between groups (group mean difference=0.14% mg/dL, SD 4.88% mg/dL; 95% CI −1.11 to 0.83; Table 3). Patients in the active control arm had a mean decrease in HbA_1c_ level of 1.42% mg/dL (SD 2.18% mg/dL; 95% CI 0.82-2.02), whereas patients in the intervention arm had a mean decrease in HbA_1c_ level of 1.28% mg/dL (SD 2.70% mg/dL; 95% CI 0.48-2.09), with a combined sample change of 1.36% mg/dL (SD 2.42% mg/dL; 95% CI 0.87-1.83). We found no confounders of the intervention effect in a mixed-effects model with individual participants with random intercepts and controlling for baseline HbA_1c_.

**Table 3:** The 6-month change in outcome measures (6 months minus baseline).

	Active control, mean change (SD; 95% CI)	Intervention, mean change (SD; 95% CI)	Group difference, mean change (SD; 95% CI)	Combined group, mean change (SD; 95% CI)
HbA_1c_^a,b^	−1.42 (2.18; −2.02 to −0.82)	−1.28 (2.70; −2.09 to −0.48)	−0.14 (4.88; −1.11 to 0.83)	−1.36 (2.42; −1.83 to −0.87)
BMI^a^	7.91 (33.80; −1.70 to 17.52)	3.84 (22.94; −2.97 to 10.65)	4.07 (58.27; −7.74 to 15.88)	5.96 (29.03; 0.08 to 11.84)
Systolic BP^a,c^	6.14 (30.45; 1.14 to 13.44)	10.06 (36.34; 0.72 to 20.83)	−3.91 (66.85; −16.56 to 8.74)	8.02 (33.28; 1.72 to 14.32)
Medication adherence^a^	15.73 (30.87; 7.13 to 24.32)	12.39 (31.11; 3.15 to 21.63)	3.34 (62.09; −9.11 to 15.78)	14.16 (30.87; 7.97 to 20.35)
SDSCA^d^—general diet^a^	1.55 (2.91; 0.74 to 2.36)	0.17 (2.69; −0.38 to 1.97)	0.37 (5.63; −0.75 to 1.50)	1.37 (2.80; 0.81 to 1.93)
SDSCA—specific diet^a^	0.42 (2.12; –0.17 to 1.01)	0.78 (1.93; 0.21 to 1.35)	−0.36 (1.68; −1.18 to 0.46)	0.60 (2.03; 0.10 to 0.18)
SDSCA—glucose monitoring^a^	0.91 (3.00; 0.08 to 1.75)	0.10 (2.84; −0.75 to 0.94)	0.82 (5.87; −0.36 to 1.99)	0.53 (2.94; −0.06 to 1.12)
SDSCA—foot care^a^	1.25 (2.96; 0.42 to 2.08)	1.36 (2.69; 0.56 to 2.16)	−0.11 (5.69; −1.25 to 1.03)	1.30 (2.83; 0.73 to 1.87)
SDSCA—carbohydrate spacing^a^	0.71 (4.34; −0.50 to 1.92)	0.20 (3.31; −0.80 to 1.20)	0.51 (7.82; −1.06 to 2.09)	0.47 (3.89; −0.31 to 1.26)
SDSCA—exercise^a^	0.39 (2.99; −0.44 to 1.23)	0.90 (3.14; −0.31 to 1.84)	−0.51 (6.13; −1.74 to 0.72)	0.63 (3.06; –0.02 to 1.25)
Self-efficacy^a^	0.17 (0.63; 0.00 to 0.35)	0.05 (0.69; −0.16 to 0.25)	0.12 (1.32; −0.14 to 0.39)	0.11 (0.66; −0.02 to 0.25)
DM^e^ distress score^a^	−0.65 (0.97; −0.92 to 0.38)	−0.60 (1.15; −0.94 to −0.26)	−0.05 (2.13; −0.48 to 0.37)	−0.63 (1.06; −0.84 to −0.42)
Depression (PHQ-9^f^)^a^	−3.86 (6.19; −5.60 to −2.12)	−2.33 (6.20; −4.17 to −0.49)	−1.54 (12.40; −4.04 to 0.96)	−3.13 (6.21; −4.39 to −1.88)
Quality of life^a^	9.70 (34.02; 0.93 to 18.45)	4.96 (29.90; −3.91 to 13.83)	4.74 (61.57; −7.61 to 17.08)	7.47 (32.16; 1.32 to 13.62)
Fatalism score^a^	1.44 (9.47; −1.25 to 4.13)	−0.58 (10.61; −3.73 to 2.57)	2.02 (20.07; −2.05 to 6.09)	0.47 (10.03; −1.56 to 2.50)
Supportive family behaviors^a^	−0.58 (7.99; −2.80 to 1.65)	−0.12 (9.08; −2.81 to 2.58)	−0.46 (17.07; −3.88 to 2.96)	−0.36 (8.48; −2.06 to 1.34)
Nonsupportive family behaviors^g^	−0.58 (6.25; −2.32 to 1.16)	−0.56 (6.86; −2.60 to 1.48)	−0.02 (13.11; −2.65 to 2.61)	−0.57 (6.51;−1.87 to 0.74)
DCP^h^ support needs^g^	−4.44 (10.79; −7.45 to −1.44)	−5.80 (9.05; −8.49 to −3.11)	1.36 (20.07; −2.66 to 5.39)	−5.08 (9.99; −7.08 to −3.08)
DCP support received^a^	0.08 (9.68; −2.62 to 2.77)	3.65 (9.10; 0.95 to 6.36)	−1.91 (18.87; −7.36 to 0.21)	1.76 (9.54; −0.16 to 3.67)
DCP support attitudes^a^	−0.96 (6.51; −2.77 to 0.85)	0.87 (5.73; −0.83 to 2.57)	−1.83 (12.34; −4.31 to 0.64)	−0.10 (6.20; −1.34 to 1.14)
Emotional support^a^	−0.64 (4.03; −1.82 to 0.54)	0.22 (4.01; −1.17 to 1.61)	−0.86 (8.43; −2.64 to 0.93)	−0.24 (4.04; −1.13 to 0.65)
Tangible support^a^	−0.06 (1.99; −0.65 to 0.52)	0.29 (2.02; −0.34 to 0.93)	−0.36 (4.02; −1.21 to 0.50)	0.10 (2.00; −0.32 to 0.53)
Supporter diabetes-related distress^g^	−0.45 (0.83; −0.66 to −0.24)	−0.28 (0.95; −0.54 to −0.02)	−0.17 (1.77; −0.50 to 0.15)	−0.37 (0.89; −0.54 to −0.21)

^a^Higher value indicates clinically worse value.

^b^HbA_1c_: glycated hemoglobin.

^c^BP: blood pressure.

^d^SDSCA: Summary of Diabetes Self-Care Activities.

^e^DM: diabetes mellitus.

^f^PHQ-9: Patient Health Questionnaire–9.

^g^Lower value indicates clinically worse value.

^h^DCP: Diabetes Care Profile.

### Secondary Outcome: 6- to 12-Month Maintenance of HbA_1c_ Level

Overall, the intervention and active control arms maintained improved HbA_1c_ levels, with a combined mean change of 0.06% mg/dL (SD 1.89% mg/dL; 95% CI −0.34 to 0.47) from the 6- to 12-month postintervention maintenance phase. There was no clinically meaningful difference between groups, with a mean increase in HbA_1c_ level of 0.36% mg/dL (SD 1.97% mg/dL; 95% CI −0.22 to 0.93) in the active control arm compared to a decrease of 0.29% mg/dL (SD 1.74; 95% CI −0.85 to 0.27) in the intervention arm (Table 4).

**Table 4:** The 12-month change in outcome measures (12 months minus 6 months).

	Active control, mean change (SD; 95% CI)	Intervention, mean change (SD; 95% CI)	Group difference, mean change (SD; 95% CI)	Combined group, mean change (SD; 95% CI)
HbA_1c_^a,b^	0.36 (1.97; −0.22 to 0.93)	−0.29 (1.74; −0.85 to 0.27)	0.65 (3.76; −0.16 to 1.45)	0.06 (1.89; −0.34 to 0.47)
BMI^b^	3.38 (22.51; −3.38 to 10.14)	−4.02 (24.88; −12.31 to 4.28)	7.40 (47.44;−3.03 to 17.82)	0.44 (23.74; −5.17 to 5.26)
Systolic BP^b,c^	4.95 (31.81; −5.09 to 14.99)	−2.09 (37.46; −14.96 to 10.78)	7.04 (69.26; −8.79 to 22.87)	1.71 (34.47; −6.17 to 9.59)
Medication adherence^b^	−1.74 (18.92; −7.36 to 3.88)	−3.22 (22.89; −10.54 to 4.10)	1.48 (41.81; −7.48 to 10.45)	−2.43 (20..75; −6.88 to 2.02)
SDSCA^d^—general diet^b^	−0.23 (2.50; −0.97 to 0.50)	−0.96 (2.74; −1.84 to −0.09)	0.73 (5.25; −0.39 to 1.84)	−0.57 (2.62; −1.13 to −0.01)
SDSCA—specific diet^b^	−0.29 (1.85; −0.83 to 0.26)	−0.45 (1.85; −1.04 to 0.14)	0.16 (3.71; −0.63 to 0.95)	−0.36 (1.84; −0.75 to 0.03)
SDSCA—glucose monitoring^b^	0.66 (3.19; −0.28 to 1.60)	0.18 (2.40; −0.59 to 0.94)	0.48 (5.72; −0.73 to 1.70)	0.43 (2.84; −1.70 to 1.04)
SDSCA—foot care^b^	0.81 (2.64; 0.03 to 1.59)	−0.01 (1.93; −0.63 to 0.61)	0.82 (4.70; −0.18 to 1.82)	0.43 (2.36; −0.07 to 0.93)
SDSCA—carbohydrate spacing^b^	−0.11 (4.51; −1.43 to 1.21)	−0.31 (3.89; −1.57 to 0.95)	0.20 (8.52; −1.63 to 2.03)	−0.20 (4.22; −1.10 to 0.71)
SDSCA—exercise^b^	−0.11 (2.57; −0.98 to 0.77)	−0.81 (2.88; −1.73 to 0.11)	0.71 (5.88; −0.55 to 1.96)	−0.43 (2.94; −1.06 to 0.19)
Self-efficacy^b^	−0.13 (0.49; −0.27 to 0.02)	0.03 (0.54; −0.14 to 0.21)	−0.16 (1.03; −0.38 to 0.06)	−0.05 (0.53; −0.16 to 0.06)
DM^e^ distress score^b^	−0.06 (0.92; −0.33 to 0.21)	0.01 (0.71; −0.22 to 0.23)	−0.07 (1.67; −0.42 to 0.29)	−0.03 (0.83; −0.21 to 0.15)
Depression (PHQ-9^f^)^b^	0.18 (6.86; −1.88 to 2.24)	0.13 (5.17; −1.53 to 1.78)	0.05 (12.27; −2.59 to 2.70)	0.15 (6.09; −1.16 to 1.47)
Quality of life^b^	−0.09 (31.47; −10.07 to 9.90)	−6.30 (29.86; −15.86 to 3.26)	6.21 (64.60; −7.56 to 19.99)	−2.94 (30.66; −9.80 to 3.91)
Fatalism score^b^	1.30 (8.97; −1.43 to 4.02)	1.20 (8.51; −4.00 to 1.59)	−0.10 (17.56; −3.96 to 3.76)	−1.26 (8.70; −3.17 to 0.66)
Supportive family behaviors^b^	−0.70 (7.87; −3.02 to 1.61)	−0.98 (9.32; −2.01 to 3.96)	−0.27 (7.19; −3.94 to 3.39)	0.83 (8.52; −0.99 to 2.64)
Nonsupportive family behaviors^g^	0.85 (6.10; −0.94 to 2.64)	1.13 (6.93; −1.09 to 3.35)	−0.28 (13.03; −3.06 to 2.50)	0.98 (6.46; −0.40 to 2.36)
DCP^h^ support needs^g^	0.39 (8.17; −2.01 to 2.78)	−0.40 (10.31; −3.70 to 2.90)	0.78 (18.48; −3.16 to 4.72)	0.02 (9.17; −1.93 to 1.98)
DCP support received^b^	1.38 (7.54; −0.83 to 3.60)	−2.70 (8.17; −5.31 to −0.09)	4.08 (15.73; 0.73 to 7.4)	−0.49 (8.16; −2.21 to 1.22)
DCP support attitudes^b^	0.77 (4.56; −0.57 to 2.11)	−0.08 (4.01; −1.36 to 1.21)	0.84 (8.67; −1.01 to 2.69)	0.38 (4.31; −0.54 to 1.30)
Emotional support^b^	0.48 (4.02; −0.72 to 1.68)	−0.69 (4.41; −1.99 to 0.61)	1.17 (8.07; −0.57 to 2.91)	−0.06 (4.20; −0.93 to 0.81)
Tangible support^b^	0.36 (2.17; −0.28 to 0.10)	−0.48 (2.08; −1.14 to 0.19)	0.84 (4.27; −0.07 to 1.75)	−0.02 (2.16;−0.48 to 0.44)

^a^HbA_1c_: glycated hemoglobin.

^b^Lower value indicates clinically worse value.

^c^BP: blood pressure.

^d^SDSCA: Summary of Diabetes Self-Care Activities.

^e^DM: diabetes mellitus.

^f^PHQ-9: Patient Health Questionnaire–9.

^g^Higher value indicates clinically worse value.

^h^DCP: Diabetes Care Profile.

### Secondary Outcome: Diabetes Self-Care Behaviors

The intervention and active control conditions were similarly efficacious in improving self-care behaviors. The 2 groups had similar changes in all self-care measures (Tables 3 and 4). We examined diabetes self-care behavior outcomes using the mixed-effects modeling described previously, allowing for random intercepts by individual patient. We found patient language preference to be a substantial confounder of intervention effect on general diet plan adherence and on disease-specific diet plan adherence, but controlling for this in a regression model did not change the group difference in intervention efficacy on self-care behaviors in the mHealth support arm versus the active control arm.

### Subgroup Analyses of Primary Outcome: 0- to 6-Month Efficacy of mHealth Support Intervention Versus Non-mHealth Standard Social Support on HbA_1c_ Level

Among patients with a new diagnosis of diabetes, intervention group patients improved their HbA_1c_ level to a greater degree than patients in the active control arm (mean between-group difference=1.96% mg/dL, SD 9.59% mg/dL; 95% CI −3.81 to −0.125; *P*=.04). We found no differences in intervention effects at 0 to 6 months based on subgroups of sex, race and ethnicity, language preference, health literacy, baseline frequency of mobile technology use, physical proximity to supporters, or baseline social support.

### Subgroup Analyses of Secondary Outcome: 6- to 12-Month Maintenance of HbA_1c_ Level

In the same prespecified subgroup analysis as that of the 0- to 6-month time frame, we found that, among patients with a new diagnosis of diabetes, intervention group patients improved their HbA_1c_ level to a greater degree than patients in the active control arm, with a predicted between-group difference of 2.4% mg/dL (SD 10.15% mg/dL; 95% CI −4.33 to −0.47; *P*=.002) at 12 months. We also found that patients who preferred SMS text messages in English maintained a better glycemic control through the mHealth social support intervention compared to the active control arm, with a predicted between-group difference of 2.53% mg/dL (SD 8.50% mg/dL; 95% CI −4.15 to −0.91; *P*=.02) at 12 months. We found no differences in HbA_1c_ level at 6 to 12 months based on sex, race and ethnicity, health literacy, baseline frequency of mobile technology use, physical proximity to supporters, and baseline social support.

### Safety

The study group had a low adverse event profile. One patient’s death due to urosepsis in the intervention arm during the postintervention maintenance phase was determined to be unrelated to the intervention after review by the local institutional review board. Severe hypoglycemic events (blood glucose of <70 mg/dL) at 6 months were self-reported in 20% (9/46) of intervention arm patients reporting at least one episode during the intervention phase and 37% (19/52) of active control arm patients reporting at least one hypoglycemic event during the intervention phase (*P*=.05).

## Discussion

### Principal Findings

In this study of augmenting existing social support via mHealth for the improvement in diabetes of safety-net ED patients, we found that all patients improved their glycemic control and self-management behaviors to a clinically significant degree. In the overall between-group analysis, we did not find that the mHealth social support intervention provided additional benefit over an active control condition of a pamphlet-based support curriculum sent to supporters. Both groups showed significant HbA_1c_ level improvements (combined group change=1.36% mg/dL, SD 2.42% mg/dL; 95% CI 0.87-1.83), with no group difference (group mean difference=0.14% mg/dL, SD 4.88; 95% CI −1.11 to 0.83) at 6 months. At 12 months, both groups maintained their improved HbA_1c_ levels, with a combined mean change from 6 months of 0.06% mg/dL (SD 1.89% mg/dL; 95% CI −0.34 to 0.47) and no clinically meaningful difference between groups. No differences were observed in safety events. In subgroup analyses, patients recently diagnosed with diabetes in the mHealth social support group improved their glycemic control to a greater degree compared to the standard social support group (between-group difference of 1.96% mg/dL, SD 9.59% mg/dL; 95% CI −3.81 to −0.125; *P*=.04)

### Comparison to Previous Literature

In previous studies, we found similar improvements in glycemic control, self-management behaviors, psychological outcomes, and social support measures to those found between the intervention and active control groups in our study. A previous pooled analyses of mHealth interventions to improve diabetes self-management showed mean HbA_1c_ level improvements of 0.5% across types of diabetes and mHealth modalities [33], whereas a previous meta-analysis of 9 traditional social support interventions for diabetes self-management showed an improvement in HbA_1c_ level of 0.25% at 3 months (95% CI −0.40 to −0.11) [34]. There is not sufficient literature to generate pooled estimates of mHealth-/eHealth-based social support for diabetes self-management [35]. As there was no placebo or sham message control group in this trial, the potential benefit of the patient-focused TExT-MED plus FANS social support augmentation via mHealth may be subject to a floor effect, with minimal improvement possible after the patient-focused intervention. In this study, we found a combined group HbA_1c_ level improvement of 1.36% mg/dL (SD 2.42% mg/dL; 95% CI 0.87-1.83) and maintenance of that improvement at 12 months with a washout period change of 0.06% (SD 1.89% mg/dL; 95% CI −0.47 to 0.34). The larger improvement in HbA_1c_ levels found in this study compared with previous literature is encouraging, suggesting that the addition of social supporters to an mHealth diabetes program, either via mHealth or standard methods, has the potential to improve the long-term health outcomes of socially vulnerable patients. In addition, we found that the intervention group reported fewer instances of hypoglycemia, an important safety outcome. mHealth social support augmentation has high potential to be translated to a system-wide intervention given the possibility of remote self-enrollment by patients and supporters, automated delivery, and minimal provider time required.

Patients who benefitted the most from the mHealth-augmented social support from the FANS intervention over the active control non-mHealth support were patients who were diagnosed with diabetes in the year before enrollment, highlighting the importance of activating social support when establishing strong self-management behaviors early in a diagnosis. Diabetes and other chronic medical conditions require complex lifestyle changes for the patient and their family members. Patients and family members may be largely unfamiliar with the best self-management practices [36-38]. A Cochrane Review analysis of a diabetes self-management education intervention versus standard care for patients with newly diagnosed diabetes showed mean differences in HbA_1c_ levels (−0.21%, 95% CI −0.38 to −0.04) 12 months after the initial intervention that consisted of educational materials [39]. National and international guidelines recommend that diabetes self-management education and support be provided to patients with diabetes upon their initial diagnosis, and this benefit is covered by many medical insurers [40,41]. However, <10% of patients receive this training internationally, with even lower rates in the United States and in underresourced environments [42-44]. In addition, there are disparities in barriers to attendance to these trainings by type of medical insurance, socioeconomic status, language, and mental health conditions [42,45,46]. Educational interventions for those with newly diagnosed diabetes need to reach patients and their families at the critical time when their health behaviors require drastic change. mHealth-based training that incorporates family members and close friends, such as TExT-MED+FANS, can overcome these barriers.

Despite our efforts to specifically address the needs of a predominantly Spanish-speaking population at our ED, including careful translation of messages and extensive pretesting for cultural and linguistic congruency [12], patients who preferred SMS text messages in English maintained their improvements in glycemic control to a greater degree. This language disparity speaks to the continued obstacles that Spanish-speaking and other non–English-speaking patients experience in accessing providers who are culturally competent and able to speak their preferred language, resulting in poor health outcomes. Spanish speakers in the United States are disproportionately burdened by type 2 diabetes compared to their English-speaking counterparts and face increased barriers to self-management support, especially when they have a non–language-congruent primary health care provider [47-49]. However, Spanish-speaking groups have benefited from mHealth interventions—the Spanish-speaking and medically underserved patients who enrolled in the Vida Health Diabetes Management Program (a novel, culturally adapted, Spanish-language mHealth diabetes program for glycemic control based out of continuity care clinics) showed an impressive decrease in HbA_1c_ levels of −1.23% at 1 year after enrollment [50]. Our initial TExT-MED patient-focused curriculum showed a 0.8% decrease in HbA_1c_ levels among Spanish-speaking ED patients at 6 months after enrollment [9]. The critical link in addressing language and cultural barriers to adequate continuity of care for diabetes management may not be fully addressed in individual-level interventions such as SMS text messages; integration into patient-centered medical homes may be required to improve patient outcomes in linguistically underserved communities.

A relatively unique feature of this study is the requirement that all patients select a family member or friend to be a supporter. Several trials of social support have shown improvements in diabetes behaviors and glycemic control when a patient elects to enroll a supporter or informal caregiver compared to patients who do not select a supporter [51-53]. However, having higher baseline social support has been associated with improved diabetes control and self-management behaviors in cross-sectional studies [54-56]. One diabetes mHealth social support pilot intervention study for physical activity in a similar population also restricted enrollment to patients who had an available supporter and showed increased perceived social support for the social support arm but no difference in physical activity recorded by a pedometer [57]. By requiring a supporter for all patient participants, we can better understand how augmenting social support adds to patient-focused interventions rather than measuring possible confounding by having enough baseline support to be able to identify a supporter. In our context, we found a comparable benefit between the mHealth-augmented social support intervention and active control condition with a non-mHealth support curriculum when added to the patient-focused curriculum. Importantly, those patients who did not continue with the entire intervention and analysis phase held negative attitudes regarding their baseline social support compared to participants who completed the study. Improvements observed in both groups may reflect patients who most enjoyed having a family member involved, which might overestimate the intervention efficacy in either the mHealth-augmented or traditional social support arms. The method of including a social support person may only need well-designed educational mailing on how to best support a loved one with diabetes and be targeted to patients who have existing strong social support relationships to activate.

### Limitations

Despite the importance of this study in this high-need population, there are several limitations. First, there was no true control group with no activation of supporters, making it difficult to determine what improvements were solely due to the patient messages versus either the mHealth or standard method of social support activation. This would underestimate the overall effect of the TExT-MED+FANS intervention. Assessing the *dose* of messaging was also difficult as there were no messages that bounced back and participants used their own devices, limiting the ability to determine how many messages were actually read. This decision to use participants’ own phones and universally compatible SMS text messaging was an important decision to maintain the pragmatic nature of this trial. We attempted a self-report measure of receipt of messages, but all patients and supporters received at least some messages and were not able to quantify the actual number. We are not able to stratify estimates of efficacy by intervention dose. In addition, we did not include a social desirability measure in baseline data, which may impact the self-reported behavior measures. However, given that there was no inactive control group, this likely had limited impact on estimates of between-group differences. Health behaviors were measured through self-report; the use of remote pill monitoring, mobile-connected pedometers, and extensive diet records was not possible and limited by the pragmatic nature of this trial. Studies with Wi-Fi-enabled pill counters and pedometers or photograph-captured food diaries would be more accurate but would have more limited data on effectiveness. The population of this study was constrained to ED patients with an HbA_1c_ level of >8.5 mg/dL, which limits generalizability to primary care populations and patients with a more modest need for improvement in their diabetes management. Of note, of 4000 potentially eligible patients, only 166 (4.15%) were enrolled; patients with sufficient technological capacity to engage in mHealth and enough baseline social support to identify and enroll a supporter may have an advantage over the average source population patient. A potentially serious limitation is the significant loss to follow-up in this highly transient patient population. We attempted daily phone calls and SMS text messages, used alternate contact numbers, and also collected values for the primary outcome (HbA_1c_ level) if available in the outcome time window. If the study had been limited to patients who had regular interaction with the health care system and more clinical data to draw from, we would have had a more complete dataset for endpoint measurements. However, we would miss the information from this highly vulnerable population with irregular access to care and infrequent HbA_1c_ measurements. The higher-than-anticipated loss to follow-up rate may have impacted the study’s power to detect a difference between the groups. However, the patients who completed the trial were generally similar to those who did not, with the exception that patients who left the study had more negative perceived attitudes toward social support than those who completed the study, potentially increasing the estimated effect of the FANS curriculum via the mHealth or standard approaches as those who stayed in the study may have had higher social support at baseline.

### Conclusions

In this randomized controlled trial of an mHealth-augmented social support curriculum added to an existing patient-focused mHealth program in ED patients with diabetes, engagement of family members via mHealth resulted in improved HbA_1c_ levels, similar to the active control condition of the same patient-focused mHealth program using a pamphlet-based support curriculum sent to family members. The TExT-MED mHealth intervention for patients with or without an mHealth-augmented social support component has the potential to improve the long-term health outcomes of these vulnerable patients and has high potential to be translated to a system-wide intervention given the possibility of remote self-enrollment by patients. Patients who were newly diagnosed with diabetes may have benefited the most from mHealth-augmented social support, with greater and more persistent improvements in glycemic control. Our exploratory findings that the activation of social support through mHealth is most helpful in patients with newly diagnosed diabetes suggest that the first years of a diabetes diagnosis are the period when family members and friends are the most *activable* via mHealth delivery of support person training.

## Data Availability

The datasets generated or analyzed during this study are not publicly available due institutional regulations but are available from the corresponding author on reasonable request.

## Appendix

Multimedia Appendix 1 Baseline characteristics—those lost to follow-up versus those who completed the 6-month assessment.

Multimedia Appendix 2 CONSORT-eHEALTH checklist (V 1.6.1).

## Abbreviations

ED: emergency department
FANS: Family and Friends Network Support
HbA_1c_: glycated hemoglobin
HIPAA: Health Insurance Portability and Accountability Act
mHealth: mobile health
TExT-MED: Trial to Examine Text Messaging in Emergency Department Patients With Diabetes
