# Supplementary material for: mHealth Social Support Versus Standard Support for Diabetes Management in Safety-Net Emergency Department Patients: Randomized Phase-III Trial
Source: JMIR Diabetes. 2025 Apr 23;10:e56934. doi: 10.2196/56934 (PMC12059508; doi:10.2196/56934)
Supplement: Multimedia Appendix 1 [file diabetes_v10i1e56934_app1.docx]

| Appendix Table 1: Baseline Characteristics: Lost To Follow Up Vs Completed Six Month Assessment | | | | |
| --- | --- | --- | --- | --- |
| Measure | | Completed Follow-Up  Mean (95% CI) or % | Loss to Follow-Up Mean (95% CI) or % | Group Difference  Mean (95% CI) |
| **A1C^a^** | | 10.86 (10.50 to 11.21) | 10.82 (10.44 to 11.20) | 0.34 (-0.50 to 0.54) |
| **Race/Ethnicity** | **Latino** | 92.99% | 91.50% |  |
| **Non-Hispanic white** | | 1.08% | 1.31% |  |
| **Asian** | | 1.08% | 1.31% |  |
| **Black** | | 4.85% | 5.88% |  |
| **Foreign Born** | | 78.92% | 77.5% |  |
| **English Languge preferred** | | 29.38% | 30.72% |  |
| **Low health literacy** | | 53.91% | 55.56% |  |
| **BMI^a^** | | 30.21 (28.67 to 31.75) | 29.87 (28.01 to 21.73) | 0.34 (-2.06 to 2.74) |
| **Age** | | 48.80 (46.80 to 50.80) | 45.78 (43.13 to 48.43) | 3.01 (-0.23 to 6.26) |
| **Quality of Life^a^** | | 59.48 (53.84 to 65.12) | 60.82 (54.06 to 67.57) | -1.34 (-10.12 to 7.45) |
| **Marin Acculturation** | | 1.90 (1.68 to 2.12) | 2.17 (1.86 to 2.49) | -0.27 (-0.64 to 0.10) |
| **DM distress score ^b^** | | 2.55 (1.01 to 2.75) | 2.37 (2.11 to 2.63) | 0.18 (-0.14 to 0.50) |
| **Fatalism score^b^** | | 35.48 (33.47 to 37.49) | 34.14 (31.80 to 36.48) | 1.34 (-1.76 to 4.44) |
| **Medication Adherence^a^** | | 66.52 (60.73 to 72.31) | 66.71 (59.19 to 74.21) | -0.18 (-9.50 to 9.14) |
| **SDSCA: general diet^a^** | | 3.33 (2.82 to 3.83) | 3.08 (2.50 to 3.67) | 0.24 (-0.53 to 1.02) |
| **SDSCA: specific diet^a^** | | 3.9 (3.53 to 4.27) | 3.82 (3.34 to 4.30) | 0.08 (-0.51 to 0.68) |
| **SDSCA: glucose monitoring^a^** | | 2.41 (1.85 to 2.96) | 3.02 (2.26 to 3.78) | -0.62 (-1.53 to 0.29) |
| **SDSCA: foot care^a^** | | 4.33 (3.75 to 4.90) | 3.66 (2.94 to 4.38) | 0.67 (-0.25 to 1.58) |
| **SDSCA: carb spacing^a^** | | 3.86 (2.36 to 3.36) | 2.92 (2.28 to 3.57) | -0.64 (-0.87 to 0.74) |
| **SDSCA: exercise^a^** | | 2.49 (1.97 to 3.01) | 2.44 (1.82 to 3.06) | 0.05 (-0.76 to 0.86) |
| **Supportive Family Behaviors^a^** | | 24.04 (22.25 to 25.83) | 23.68 (21.54 to 25.82) | 0.35 (-2.43 to 3.14) |
| **Non-Supportive Family Behaviors ^b^** | | 18.31 (16.97 to 19.65) | 18.10 (16.52 to 19.68) | 0.21 (-1.87 to 2.28) |
| **Support needs**  **Diabetes Care Profile ^b^** | | 24.1 (22.70 to 25.50) | 22.88 (20.88 to 24.88) | 1.22 (-1.13 to 3.57) |
| **Support received**  **Diabetes Care Profile^a^** | | 18.57 (16.74 to 20.40) | 18.52 (16.43 to 20.60) | 0.05 (-2.74 to 2.85) |
| **Support attitudes Diabetes Care Profile^a^** | | 7.36 (6.46 to 8.26) | 5.08 (3.66 to 6.50) | 2.28 (-0.70 to 3.87) |
| **Support attitudes: positive sub-score Diabetes Care Profile^a^** | | 4.01 (3.74 to 4.29) | 3.94 (3.61 to 4.28) | 0.07 (-0.36 to 0.50) |
| **Support attitudes: Negative sub-score Diabetes Care Profile ^b^** | | 1.56 (1.36 to 1.76) | 2.25 (1.95 to 2.55) | -0.69 (-1.03 to -0.35) |
| **Emotional support^a^** | | 13.77 (13.04 to 14.49) | 13.95 (13.22 to 14.68) | -0.18 (-1.24 to 0.87) |
| **Tangible support^a^** | | 6.94 (6.59 to 7.29) | 7.05 (6.62 to 7.48) | -0.10 (-0.65 to 0.44) |
| **^a^ Higher value indicates clinically worse value**  **^b^ Lower value indicates clinically worse value** | | | | |
