# Supplementary material for: mHealth Social Support Versus Standard Support for Diabetes Management in Safety-Net Emergency Department Patients: Randomized Phase-III Trial
Source: JMIR Diabetes. 2025 Apr 23;10:e56934. doi: 10.2196/56934 (PMC12059508; doi:10.2196/56934)
Supplement: Multimedia Appendix 2 [file diabetes_v10i1e56934_app2.pdf]

CONSORT-EHEALTH (V 1.6.1) - Submission/Publication Form

The CONSORT-EHEALTH checklist is intended for authors of randomized trials evaluating web-based and Internet-based applications/interventions, including mobile interventions, electronic games (incl multiplayer games), social media, certain telehealth applications, and other interactive and/or networked electronic applications. Some of the items (e.g. all subitems under item 5 - description of the intervention) may also be applicable for other study designs.

The goal of the CONSORT EHEALTH checklist and guideline is to be
a) a guide for reporting for authors of RCTs,
b) to form a basis for appraisal of an ehealth trial (in terms of validity)

CONSORT-EHEALTH items/subitems are MANDATORY reporting items for studies published in the Journal of Medical Internet Research and other journals / scientific societies endorsing the checklist.

Items numbered 1., 2., 3., 4a., 4b etc are original CONSORT or CONSORT-NPT (non-pharmacologic treatment) items.
Items with Roman numerals (i., ii, iii, iv etc.) are CONSORT-EHEALTH extensions/clarifications.

As the CONSORT-EHEALTH checklist is still considered in a formative stage, we would ask that you also RATE ON A SCALE OF 1-5 how important/useful you feel each item is FOR THE PURPOSE OF THE CHECKLIST and reporting guideline (optional).

Mandatory reporting items are marked with a red *.
In the textboxes, either copy & paste the relevant sections from your manuscript into this form - please include any quotes from your manuscript in QUOTATION MARKS,
or answer directly by providing additional information not in the manuscript, or elaborating on why the item was not relevant for this study.

YOUR ANSWERS WILL BE PUBLISHED AS A SUPPLEMENTARY FILE TO YOUR PUBLICATION IN JMIR AND ARE CONSIDERED PART OF YOUR PUBLICATION (IF ACCEPTED).
Please fill in these questions diligently. Information will not be copyedited, so please use proper spelling and grammar, use correct capitalization, and avoid abbreviations.

DO NOT FORGET TO SAVE AS PDF _AND_ CLICK THE SUBMIT BUTTON SO YOUR ANSWERS ARE IN OUR DATABASE !!!

Citation Suggestion (if you append the pdf as Appendix we suggest to cite this paper in the caption):
Eysenbach G, CONSORT-EHEALTH Group
CONSORT-EHEALTH: Improving and Standardizing Evaluation Reports of Web-based and Mobile Health Interventions
J Med Internet Res 2011;13(4):e126
URL: http://www.jmir.org/2011/4/e126/
doi: 10.2196/jmir.1923
PMID: 22209829

lizburner@gmail.com Switch account

Not shared

Draft not saved

* Indicates required question

Your name *

First Last

Elizabeth Burner

Primary Affiliation (short), City, Country *

University of Toronto, Toronto, Canada

Univesiry of Southern California, Los Angeles,

Your e-mail address *

abc@gmail.com

eburner@usc.edu

Title of your manuscript *

Provide the (draft) title of your manuscript.

Outcomes of TExT-MED+FANS: a Phase III Randomized Unblinded Trial of mHealth Augmented Social Support vs Standard Social Support in Combination with a mHealth Curriculum for Safety-Net ED Patients with Diabetes

Name of your App/Software/Intervention *

If there is a short and a long/alternate name, write the short name first and add the long name in brackets.

TExT-MED+FANS (Trial to Examine Text Messa

Evaluated Version (if any)

e.g. "V1", "Release 2017-03-01", "Version 2.0.27913"

Your answer

Language(s) *

What language is the intervention/app in? If multiple languages are available, separate by comma (e.g. "English, French")

English, Spanish

URL of your Intervention Website or App

e.g. a direct link to the mobile app on app in appstore (itunes, Google Play), or URL of the website. If the intervention is a DVD or hardware, you can also link to an Amazon page.

Your answer

URL of an image/screenshot (optional)

Your answer

Accessibility *

Can an enduser access the intervention presently?

- ○ access is free and open
- ○ access only for special usergroups, not open
- ◉ access is open to everyone, but requires payment/subscription/in-app purchases
- ○ app/intervention no longer accessible
- ○ Other:

Primary Medical Indication/Disease/Condition *

e.g. "Stress", "Diabetes", or define the target group in brackets after the condition, e.g. "Autism (Parents of children with)", "Alzheimers (Informal Caregivers of)"

Diabetes

Primary Outcomes measured in trial *

comma-separated list of primary outcomes reported in the trial

glycosylated hemoglobin

Secondary/other outcomes

Are there any other outcomes the intervention is expected to affect?

Your answer

Recommended "Dose" *

What do the instructions for users say on how often the app should be used?

- [x] Approximately Daily
- [ ] Approximately Weekly
- [ ] Approximately Monthly
- [ ] Approximately Yearly
- [ ] "as needed"
- [ ] Other: ____

Approx. Percentage of Users (starters) still using the app as recommended after 3 months *

- ( ) unknown / not evaluated
- ( ) 0-10%
- ( ) 11-20%
- ( ) 21-30%
- ( ) 31-40%
- ( ) 41-50%
- ( ) 51-60%
- (x) 61-70%
- ( ) 71%-80%
- ( ) 81-90%
- ( ) 91-100%
- ( ) Other: ______

Overall, was the app/intervention effective? *

- ○ yes: all primary outcomes were significantly better in intervention group vs control
- ○ partly: SOME primary outcomes were significantly better in intervention group vs control
- ◉ no statistically significant difference between control and intervention
- ○ potentially harmful: control was significantly better than intervention in one or more outcomes
- ○ inconclusive: more research is needed
- ○ Other: ____________________

Article Preparation Status/Stage *

At which stage in your article preparation are you currently (at the time you fill in this form)

- ○ not submitted yet - in early draft status
- ○ not submitted yet - in late draft status, just before submission
- ◉ submitted to a journal but not reviewed yet
- ○ submitted to a journal and after receiving initial reviewer comments
- ○ submitted to a journal and accepted, but not published yet
- ○ published
- ○ Other: ____________________

Journal *

If you already know where you will submit this paper (or if it is already submitted), please provide the journal name (if it is not JMIR, provide the journal name under "other")

- ○ not submitted yet / unclear where I will submit this
- ○ Journal of Medical Internet Research (JMIR)
- ○ JMIR mHealth and UHealth
- ○ JMIR Serious Games
- ○ JMIR Mental Health
- ○ JMIR Public Health
- ○ JMIR Formative Research
- ◉ Other JMIR sister journal
- ○ Other: ______

Is this a full powered effectiveness trial or a pilot/feasibility trial? *

- ○ Pilot/feasibility
- ◉ Fully powered

Manuscript tracking number *

If this is a JMIR submission, please provide the manuscript tracking number under "other" (The ms tracking number can be found in the submission acknowledgement email, or when you login as author in JMIR. If the paper is already published in JMIR, then the ms tracking number is the four-digit number at the end of the DOI, to be found at the bottom of each published article in JMIR)

- ○ no ms number (yet) / not (yet) submitted to / published in JMIR
- ● Other: JD ms#56934

TITLE AND ABSTRACT

1a) TITLE: Identification as a randomized trial in the title

1a) Does your paper address CONSORT item 1a? *

I.e does the title contain the phrase "Randomized Controlled Trial"? (if not, explain the reason under "other")

- ● yes
- ○ Other:

1a-i) Identify the mode of delivery in the title

Identify the mode of delivery. Preferably use “web-based” and/or “mobile” and/or “electronic game” in the title. Avoid ambiguous terms like “online”, “virtual”, “interactive”. Use “Internet-based” only if Intervention includes non-web-based Internet components (e.g. email), use “computer-based” or “electronic” only if offline products are used. Use “virtual” only in the context of “virtual reality” (3-D worlds). Use “online” only in the context of “online support groups”. Complement or substitute product names with broader terms for the class of products (such as “mobile” or “smart phone” instead of “iphone”), especially if the application runs on different platforms.

	1	2	3	4	5	
subitem not at all important	○	○	◉	○	○	essential

Clear selection

Does your paper address subitem 1a-i? *

Copy and paste relevant sections from manuscript title (include quotes in quotation marks "like this" to indicate direct quotes from your manuscript), or elaborate on this item by providing additional information not in the ms, or briefly explain why the item is not applicable/relevant for your study

title is already very long

1a-ii) Non-web-based components or important co-interventions in title

Mention non-web-based components or important co-interventions in title, if any (e.g., “with telephone support”).

	1	2	3	4	5	
subitem not at all important	○	○	◉	○	○	essential

Clear selection

Does your paper address subitem 1a-ii?

Copy and paste relevant sections from manuscript title (include quotes in quotation marks "like this" to indicate direct quotes from your manuscript), or elaborate on this item by providing additional information not in the ms, or briefly explain why the item is not applicable/relevant for your study

title is already very long

1a-iii) Primary condition or target group in the title

Mention primary condition or target group in the title, if any (e.g., “for children with Type I Diabetes”) Example: A Web-based and Mobile Intervention with Telephone Support for Children with Type I Diabetes: Randomized Controlled Trial

	1	2	3	4	5	
subitem not at all important	○	○	○	○	◉	essential

Clear selection

Does your paper address subitem 1a-iii? *

Copy and paste relevant sections from manuscript title (include quotes in quotation marks "like this" to indicate direct quotes from your manuscript), or elaborate on this item by providing additional information not in the ms, or briefly explain why the item is not applicable/relevant for your study

"Outcomes of TExT-MED+FANS: a Phase III Randomized Unblinded Trial of mHealth Augmented Social Support vs Standard Social Support in Combination with a mHealth Curriculum for Safety-Net ED Patients with Diabetes"

1b) ABSTRACT: Structured summary of trial design, methods, results, and conclusions

NPT extension: Description of experimental treatment, comparator, care providers, centers, and blinding status.

1b-i) Key features/functionalities/components of the intervention and comparator in the METHODS section of the ABSTRACT

Mention key features/functionalities/components of the intervention and comparator in the abstract. If possible, also mention theories and principles used for designing the site. Keep in mind the needs of systematic reviewers and indexers by including important synonyms. (Note: Only report in the abstract what the main paper is reporting. If this information is missing from the main body of text, consider adding it)

	1	2	3	4	5	
subitem not at all important	○	○	○	○	◉	essential

Clear selection

Does your paper address subitem 1b-i? *

Copy and paste relevant sections from the manuscript abstract (include quotes in quotation marks "like this" to indicate direct quotes from your manuscript), or elaborate on this item by providing additional information not in the ms, or briefly explain why the item is not applicable/relevant for your study

"Patients with A1C≥8.5%mg/dL and a text-capable phone were recruited during their ED visit. They received 6 months of the TExT-MED diabetes self-management mHealth program. Supporters were randomized to receive either 1) Family and Friends Network Support (FANS) intervention: daily text messages guiding supporters to provide diabetes-related social support or 2) an active control: pamphlet-augmented social support with FANS content."

1b-ii) Level of human involvement in the METHODS section of the ABSTRACT

Clarify the level of human involvement in the abstract, e.g., use phrases like “fully automated” vs. “therapist/nurse/care provider/physician-assisted” (mention number and expertise of providers involved, if any). (Note: Only report in the abstract what the main paper is reporting. If this information is missing from the main body of text, consider adding it)

	1	2	3	4	5	
subitem not at all important	○	○	○	○	◉	essential

Clear selection

Does your paper address subitem 1b-ii?

Copy and paste relevant sections from the manuscript abstract (include quotes in quotation marks "like this" to indicate direct quotes from your manuscript), or elaborate on this item by providing additional information not in the ms, or briefly explain why the item is not applicable/relevant for your study

"Patients with A1C≥8.5%mg/dL and a text-capable phone were recruited during their ED visit. They received 6 months of the TExT-MED diabetes self-management mHealth program. Supporters were randomized to receive either 1) Family and Friends Network Support (FANS) intervention: daily text messages guiding supporters to provide diabetes-related social support or 2) an active control: pamphlet-augmented social support with FANS content."

1b-iii) Open vs. closed, web-based (self-assessment) vs. face-to-face assessments in the METHODS section of the ABSTRACT

Mention how participants were recruited (online vs. offline), e.g., from an open access website or from a clinic or a closed online user group (closed usergroup trial), and clarify if this was a purely web-based trial, or there were face-to-face components (as part of the intervention or for assessment). Clearly say if outcomes were self-assessed through questionnaires (as common in web-based trials). Note: In traditional offline trials, an open trial (open-label trial) is a type of clinical trial in which both the researchers and participants know which treatment is being administered. To avoid confusion, use “blinded” or “unblinded” to indicated the level of blinding instead of “open”, as “open” in web-based trials usually refers to “open access” (i.e. participants can self-enrol). (Note: Only report in the abstract what the main paper is reporting. If this information is missing from the main body of text, consider adding it)

	1	2	3	4	5	
subitem not at all important	○	○	○	○	◉	essential

Clear selection

Does your paper address subitem 1b-iii?

Copy and paste relevant sections from the manuscript abstract (include quotes in quotation marks "like this" to indicate direct quotes from your manuscript), or elaborate on this item by providing additional information not in the ms, or briefly explain why the item is not applicable/relevant for your study

"A1C, self-reported diabetes self-care activities, medication adherence, and safety events were measured. "

1b-iv) RESULTS section in abstract must contain use data

Report number of participants enrolled/assessed in each group, the use/uptake of the intervention (e.g., attrition/adherence metrics, use over time, number of logins etc.), in addition to primary/secondary outcomes. (Note: Only report in the abstract what the main paper is reporting. If this information is missing from the main body of text, consider adding it)

	1	2	3	4	5	
subitem not at all important	○	○	○	○	◉	essential

Clear selection

Does your paper address subitem 1b-iv?

Copy and paste relevant sections from the manuscript abstract (include quotes in quotation marks "like this" to indicate direct quotes from your manuscript), or elaborate on this item by providing additional information not in the ms, or briefly explain why the item is not applicable/relevant for your study

"166 patients were randomized, with 97 (58%) followed at 6 months and 106 (64%) at 12 months. Both intervention groups showed significant A1C improvements (combined group change 1.36%mg/dl, (95% CI 0.87 to 1.83)), with no group difference (group mean difference = 0.14%mg/dl (95% CI 0.83 to -1.11)). No differences were observed in safety events."

1b-v) CONCLUSIONS/DISCUSSION in abstract for negative trials

Conclusions/Discussions in abstract for negative trials: Discuss the primary outcome - if the trial is negative (primary outcome not changed), and the intervention was not used, discuss whether negative results are attributable to lack of uptake and discuss reasons. (Note: Only report in the abstract what the main paper is reporting. If this information is missing from the main body of text, consider adding it)

	1	2	3	4	5	
subitem not at all important	○	○	○	○	◉	essential

Clear selection

Does your paper address subitem 1b-v?

Copy and paste relevant sections from the manuscript abstract (include quotes in quotation marks "like this" to indicate direct quotes from your manuscript), or elaborate on this item by providing additional information not in the ms, or briefly explain why the item is not applicable/relevant for your study

"Six-month change in A1C did not differ by mode of social support in persons using an existing patient-focused mHealth diabetes self-management program. Newly diagnosed diabetes patients benefiting most from mHealth augmented social support."

INTRODUCTION

2a) In INTRODUCTION: Scientific background and explanation of rationale

2a-i) Problem and the type of system/solution

Describe the problem and the type of system/solution that is object of the study: intended as stand-alone intervention vs. incorporated in broader health care program? Intended for a particular patient population? Goals of the intervention, e.g., being more cost-effective to other interventions, replace or complement other solutions? (Note: Details about the intervention are provided in “Methods” under 5)

	1	2	3	4	5	
subitem not at all important	○	○	◉	○	○	essential

Clear selection

Does your paper address subitem 2a-i? *

Copy and paste relevant sections from the manuscript (include quotes in quotation marks "like this" to indicate direct quotes from your manuscript), or elaborate on this item by providing additional information not in the ms, or briefly explain why the item is not applicable/relevant for your study

Particularly in under-resourced populations, patient focused interventions could be strengthened by adding social support for diabetes modules for loved ones of people with diabetes. Using mobile training, a patient can select anyone from their social support network, regardless of physical location to be a “FANS” (Family And friends Network Supporter). Adding this mobile social support module, TExT-MED+FANS, builds on the success of the original TExT-MED intervention by adding an emotional and highly personal touch to enhance results.

2a-ii) Scientific background, rationale: What is known about the (type of) system

Scientific background, rationale: What is known about the (type of) system that is the object of the study (be sure to discuss the use of similar systems for other conditions/diagnoses, if appropiate), motivation for the study, i.e. what are the reasons for and what is the context for this specific study, from which stakeholder viewpoint is the study performed, potential impact of findings [2]. Briefly justify the choice of the comparator.

	1	2	3	4	5	
subitem not at all important	○	○	◉	○	○	essential

Clear selection

Does your paper address subitem 2a-ii? *

Copy and paste relevant sections from the manuscript (include quotes in quotation marks "like this" to indicate direct quotes from your manuscript), or elaborate on this item by providing additional information not in the ms, or briefly explain why the item is not applicable/relevant for your study

"mHealth-based social support interventions may increase the efficacy and effectiveness of patient focused mHealth interventions for diabetes self-management. mHealth for diabetes self-management is effective, with improvements in A1C of 0.3% to 0.8%.4 Despite this, a digital divide persists, with less uptake in populations with lower social economic status and minoritized backgrounds.5 SMS-based mHealth strategies have successfully been employed in vulnerable patient populations,6 such as CareMessages7 in federally qualified health centers in Los Angeles County and REACH among low-income, primary care clinic patients in Nashville.8
Outside of primary care settings, the TExT-MED intervention was tested in safety-net Emergency Department patients.9 The original six-month, fully automated, text message-based TExT-MED curriculum is based on National Diabetes Education Program (NDEP)10 messages, adapted for text message character limits and emphasizes education and behavior changes. Intervention participants improved their A1C 0.1% compared to control participants. Particularly in under-resourced populations, patient focused interventions could be strengthened by adding social support for diabetes modules for loved ones of people with diabetes. Using mobile training, a patient can select anyone from their social support network, regardless of physical location to be a "FANS" (Family And friends Network Supporter). Adding this mobile social support module, TExT-MED+FANS, builds on the success of the original TExT-MED intervention by adding an emotional and highly personal touch to enhance results. "

2b) In INTRODUCTION: Specific objectives or hypotheses

Does your paper address CONSORT subitem 2b? *

Copy and paste relevant sections from the manuscript (include quotes in quotation marks "like this" to indicate direct quotes from your manuscript), or elaborate on this item by providing additional information not in the ms, or briefly explain why the item is not applicable/relevant for your study

mHealth-based social support interventions may increase the efficacy and effectiveness of patient focused mHealth interventions for diabetes self-management. mHealth for diabetes self-management is effective, with improvements in A1C of 0.3% to 0.8%.4 Despite this, a digital divide persists, with less uptake in populations with lower social economic status and minoritized backgrounds.5 SMS-based mHealth strategies have successfully been employed in vulnerable patient populations,6 such as CareMessages7 in federally qualified health centers in Los Angeles County and REACH among low-income, primary care clinic patients in Nashville.8
Outside of primary care settings, the TExT-MED intervention was tested in safety-net Emergency Department patients.9 The original six-month, fully automated, text message-based TExT-MED curriculum is based on National Diabetes Education Program (NDEP)10 messages, adapted for text message character limits and emphasizes education and behavior changes. Intervention participants improved their A1C 0.1% compared to control participants. Particularly in under-resourced populations, patient focused interventions could be strengthened by adding social support for diabetes modules for loved ones of people with diabetes. Using mobile training, a patient can select anyone from their social support network, regardless of physical location to be a “FANS” (Family And friends Network Supporter). Adding this mobile social support module, TExT-MED+FANS, builds on the success of the original TExT-MED intervention by adding an emotional and highly personal touch to enhance results.

METHODS

3a) Description of trial design (such as parallel, factorial) including allocation ratio

Does your paper address CONSORT subitem 3a? *

Copy and paste relevant sections from the manuscript (include quotes in quotation marks "like this" to indicate direct quotes from your manuscript), or elaborate on this item by providing additional information not in the ms, or briefly explain why the item is not applicable/relevant for your study

This study was an unblinded, randomized, parallel, active controlled intervention with 1:1 allocation ratio.

3b) Important changes to methods after trial commencement (such as eligibility criteria), with reasons

Does your paper address CONSORT subitem 3b? *

Copy and paste relevant sections from the manuscript (include quotes in quotation marks "like this" to indicate direct quotes from your manuscript), or elaborate on this item by providing additional information not in the ms, or briefly explain why the item is not applicable/relevant for your study

this does not apply to this manuscript

3b-i) Bug fixes, Downtimes, Content Changes

Bug fixes, Downtimes, Content Changes: ehealth systems are often dynamic systems. A description of changes to methods therefore also includes important changes made on the intervention or comparator during the trial (e.g., major bug fixes or changes in the functionality or content) (5-iii) and other “unexpected events” that may have influenced study design such as staff changes, system failures/downtimes, etc. [2].

	1	2	3	4	5	
subitem not at all important	○	○	○	○	◉	essential

Clear selection

Does your paper address subitem 3b-i?

Copy and paste relevant sections from the manuscript (include quotes in quotation marks "like this" to indicate direct quotes from your manuscript), or elaborate on this item by providing additional information not in the ms, or briefly explain why the item is not applicable/relevant for your study

this does not apply to this manuscript

4a) Eligibility criteria for participants

Does your paper address CONSORT subitem 4a? *

Copy and paste relevant sections from the manuscript (include quotes in quotation marks "like this" to indicate direct quotes from your manuscript), or elaborate on this item by providing additional information not in the ms, or briefly explain why the item is not applicable/relevant for your study

Patients with diabetes were screened and enrolled by surveying the ED electronic patient tracking system from July 2017 to October 2018. Inclusion criteria were age 18 or older, A1C 8.5%mg/dl or greater, and able to consent. Patients were excluded if they did not have stable ownership of a mobile phone for 30 days or more, were not able to send and receive text messages, did not read English or Spanish, or could not identify a support person who could be contacted within 2 weeks to enroll. Patients who reported type 1, type 2, or unknown type of diabetes were enrolled, as prior work with this population has shown that 30% of patients are unsure which type of diabetes they have 12.

4a-i) Computer / Internet literacy

Computer / Internet literacy is often an implicit “de facto” eligibility criterion - this should be explicitly clarified.

	1	2	3	4	5	
subitem not at all important	○	○	○	○	◉	essential

Clear selection

Does your paper address subitem 4a-i?

Copy and paste relevant sections from the manuscript (include quotes in quotation marks "like this" to indicate direct quotes from your manuscript), or elaborate on this item by providing additional information not in the ms, or briefly explain why the item is not applicable/relevant for your study

Patients with diabetes were screened and enrolled by surveying the ED electronic patient tracking system from July 2017 to October 2018. Inclusion criteria were age 18 or older, A1C 8.5%mg/dl or greater, and able to consent. Patients were excluded if they did not have stable ownership of a mobile phone for 30 days or more, were not able to send and receive text messages, did not read English or Spanish, or could not identify a support person who could be contacted within 2 weeks to enroll. Patients who reported type 1, type 2, or unknown type of diabetes were enrolled, as prior work with this population has shown that 30% of patients are unsure which type of diabetes they have 12.

4a-ii) Open vs. closed, web-based vs. face-to-face assessments:

Open vs. closed, web-based vs. face-to-face assessments: Mention how participants were recruited (online vs. offline), e.g., from an open access website or from a clinic, and clarify if this was a purely web-based trial, or there were face-to-face components (as part of the intervention or for assessment), i.e., to what degree got the study team to know the participant. In online-only trials, clarify if participants were quasi-anonymous and whether having multiple identities was possible or whether technical or logistical measures (e.g., cookies, email confirmation, phone calls) were used to detect/prevent these.

	1	2	3	4	5	
subitem not at all important	○	○	○	○	◉	essential

Clear selection

Does your paper address subitem 4a-ii? *

Copy and paste relevant sections from the manuscript (include quotes in quotation marks "like this" to indicate direct quotes from your manuscript), or elaborate on this item by providing additional information not in the ms, or briefly explain why the item is not applicable/relevant for your study

Data collection procedures, schedule, and outcome measures for patients
Patient assessments occurred at enrollment, three, six, nine, and twelve months on behavioral and psychosocial outcomes and at baseline, six, and twelve months for clinical outcomes. Trained RAs conducted in-person assessments at an office at the medical center using standardized protocols and equipment, in either English or Spanish depending on patient preference. For assessments at three and nine months, participants had the option of an in-person, mail, or phone appointment. Data was entered into the REDCap system maintained by the SC CTSI.15

4a-iii) Information giving during recruitment

Information given during recruitment. Specify how participants were briefed for recruitment and in the informed consent procedures (e.g., publish the informed consent documentation as appendix, see also item X26), as this information may have an effect on user self-selection, user expectation and may also bias results.

	1	2	3	4	5	
subitem not at all important	○	○	○	○	◉	essential

Clear selection

Does your paper address subitem 4a-iii?

Copy and paste relevant sections from the manuscript (include quotes in quotation marks "like this" to indicate direct quotes from your manuscript), or elaborate on this item by providing additional information not in the ms, or briefly explain why the item is not applicable/relevant for your study

Research assistants (RAs) explained the purpose of the study and obtained consent in the ED. Patients were aware at enrollment that the designated supporter could receive multiple text-messages per day and would be prompted to offer increased support.

4b) Settings and locations where the data were collected

Does your paper address CONSORT subitem 4b? *

Copy and paste relevant sections from the manuscript (include quotes in quotation marks "like this" to indicate direct quotes from your manuscript), or elaborate on this item by providing additional information not in the ms, or briefly explain why the item is not applicable/relevant for your study

Data collection procedures, schedule, and outcome measures for patients
Patient assessments occurred at enrollment, three, six, nine, and twelve months on behavioral and psychosocial outcomes and at baseline, six, and twelve months for clinical outcomes. Trained RAs conducted in-person assessments at an office at the medical center using standardized protocols and equipment, in either English or Spanish depending on patient preference. For assessments at three and nine months, participants had the option of an in-person, mail, or phone appointment. Data was entered into the REDCap system maintained by the SC CTSI.15

4b-i) Report if outcomes were (self-)assessed through online questionnaires

Clearly report if outcomes were (self-)assessed through online questionnaires (as common in web-based trials) or otherwise.

	1	2	3	4	5	
subitem not at all important	○	○	○	○	◉	essential

Clear selection

Does your paper address subitem 4b-i? *

Copy and paste relevant sections from the manuscript (include quotes in quotation marks "like this" to indicate direct quotes from your manuscript), or elaborate on this item by providing additional information not in the ms, or briefly explain why the item is not applicable/relevant for your study

Data collection procedures, schedule, and outcome measures for patients
Patient assessments occurred at enrollment, three, six, nine, and twelve months on behavioral and psychosocial outcomes and at baseline, six, and twelve months for clinical outcomes. Trained RAs conducted in-person assessments at an office at the medical center using standardized protocols and equipment, in either English or Spanish depending on patient preference. For assessments at three and nine months, participants had the option of an in-person, mail, or phone appointment. Data was entered into the REDCap system maintained by the SC CTSI.15

4b-ii) Report how institutional affiliations are displayed

Report how institutional affiliations are displayed to potential participants [on ehealth media], as affiliations with prestigious hospitals or universities may affect volunteer rates, use, and reactions with regards to an intervention.(Not a required item – describe only if this may bias results)

	1	2	3	4	5	
subitem not at all important	○	○	○	○	○	essential

Does your paper address subitem 4b-ii?

Copy and paste relevant sections from the manuscript (include quotes in quotation marks "like this" to indicate direct quotes from your manuscript), or elaborate on this item by providing additional information not in the ms, or briefly explain why the item is not applicable/relevant for your study

Your answer

5) The interventions for each group with sufficient details to allow replication, including how and when they were actually administered

5-i) Mention names, credential, affiliations of the developers, sponsors, and owners

Mention names, credential, affiliations of the developers, sponsors, and owners [6] (if authors/evaluators are owners or developer of the software, this needs to be declared in a “Conflict of interest” section or mentioned elsewhere in the manuscript).

	1	2	3	4	5	
subitem not at all important	○	○	○	○	◉	essential

Clear selection

Does your paper address subitem 5-i?

Copy and paste relevant sections from the manuscript (include quotes in quotation marks "like this" to indicate direct quotes from your manuscript), or elaborate on this item by providing additional information not in the ms, or briefly explain why the item is not applicable/relevant for your study

this does not apply to this study

5-ii) Describe the history/development process

Describe the history/development process of the application and previous formative evaluations (e.g., focus groups, usability testing), as these will have an impact on adoption/use rates and help with interpreting results.

	1	2	3	4	5	
subitem not at all important	○	○	○	○	○	essential

Does your paper address subitem 5-ii?

Copy and paste relevant sections from the manuscript (include quotes in quotation marks "like this" to indicate direct quotes from your manuscript), or elaborate on this item by providing additional information not in the ms, or briefly explain why the item is not applicable/relevant for your study

Patient Intervention – TExT-MED
The original six-month, fully automated, text message-based TExT-MED curriculum was previously described 9. It is based on National Diabetes Education Program (NDEP)10 messages, adapted for text message character limits (160 characters); messages emphasized education and behavior changes. The program was designed to enhance knowledge, self-efficacy and diabetes self-management . Patient messages, delivered twice daily, included 1) educational/motivational messages 2) medication reminders 3) diabetes trivia questions and 4) healthy living challenges.

Following the original TExT-MED study, Agile Health purchased, modified, and commercialized the program as MyAgileLife. This enhanced version delivers three daily messages and has a greater focus on skills such as setting goals, enabling social support, and increasing engagement. To synchronize patient and supporter messages, we used a locally modified MyAgileLife version. All patients received TExT-MED.

FANS Family member/Supporter Intervention - FANS Curriculum
The development of the FANS support curriculum is described in a separate manuscript.13 In brief, the messages were developed from National Diabetes Education Program and American Diabetes Association (ADA) recommendations, synchronizing in content and time to 2 of the 3 daily patient messages. The FANS messages focused on: 1) Instrumental support (tangible goods and actions), 2) Informational support (knowledge) and 3) Emotional support.14 Given the financial constraints the patients and family members of this population, FANS messages emphasized non-financial forms of instrumental support. One FANS message per week was an active support challenge message that encouraged patient contact, emphasized a specific support care behavior, or challenged the FANS to perform the same health behavior as the patient, and to communicate that to the patient. In total, the FANS curriculum consists of 381 messages.

5-iii) Revisions and updating

Revisions and updating. Clearly mention the date and/or version number of the application/intervention (and comparator, if applicable) evaluated, or describe whether the intervention underwent major changes during the evaluation process, or whether the development and/or content was “frozen” during the trial. Describe dynamic components such as news feeds or changing content which may have an impact on the replicability of the intervention (for unexpected events see item 3b).

	1	2	3	4	5	
subitem not at all important	○	○	○	○	◉	essential

Clear selection

Does your paper address subitem 5-iii?

Copy and paste relevant sections from the manuscript (include quotes in quotation marks "like this" to indicate direct quotes from your manuscript), or elaborate on this item by providing additional information not in the ms, or briefly explain why the item is not applicable/relevant for your study

Patient Intervention – TExT-MED
The original six-month, fully automated, text message-based TExT-MED curriculum was previously described 9. It is based on National Diabetes Education Program (NDEP)10 messages, adapted for text message character limits (160 characters); messages emphasized education and behavior changes. The program was designed to enhance knowledge, self-efficacy and diabetes self-management . Patient messages, delivered twice daily, included 1) educational/motivational messages 2) medication reminders 3) diabetes trivia questions and 4) healthy living challenges.

Following the original TExT-MED study, Agile Health purchased, modified, and commercialized the program as MyAgileLife. This enhanced version delivers three daily messages and has a greater focus on skills such as setting goals, enabling social support, and increasing engagement. To synchronize patient and supporter messages, we used a locally modified MyAgileLife version. All patients received TExT-MED.

FANS Family member/Supporter Intervention - FANS Curriculum
The development of the FANS support curriculum is described in a separate manuscript.13 In brief, the messages were developed from National Diabetes Education Program and American Diabetes Association (ADA) recommendations, synchronizing in content and time to 2 of the 3 daily patient messages. The FANS messages focused on: 1) Instrumental support (tangible goods and actions), 2) Informational support (knowledge) and 3) Emotional support.14 Given the financial constraints the patients and family members of this population, FANS messages emphasized non-financial forms of instrumental support. One FANS message per week was an active support challenge message that encouraged patient contact, emphasized a specific support care behavior, or challenged the FANS to perform the same health behavior as the patient, and to communicate that to the patient. In total, the FANS curriculum consists of 381 messages.

5-iv) Quality assurance methods

Provide information on quality assurance methods to ensure accuracy and quality of information provided [1], if applicable.

	1	2	3	4	5	
subitem not at all important	○	○	○	○	○	essential

Does your paper address subitem 5-iv?

Copy and paste relevant sections from the manuscript (include quotes in quotation marks "like this" to indicate direct quotes from your manuscript), or elaborate on this item by providing additional information not in the ms, or briefly explain why the item is not applicable/relevant for your study

this does not apply to this study

5-v) Ensure replicability by publishing the source code, and/or providing screenshots/screen-capture video, and/or providing flowcharts of the algorithms used

Ensure replicability by publishing the source code, and/or providing screenshots/screen-capture video, and/or providing flowcharts of the algorithms used. Replicability (i.e., other researchers should in principle be able to replicate the study) is a hallmark of scientific reporting.

	1	2	3	4	5	
subitem not at all important	○	○	○	○	○	essential

Does your paper address subitem 5-v?

Copy and paste relevant sections from the manuscript (include quotes in quotation marks "like this" to indicate direct quotes from your manuscript), or elaborate on this item by providing additional information not in the ms, or briefly explain why the item is not applicable/relevant for your study

this does not apply to this study

5-vi) Digital preservation

Digital preservation: Provide the URL of the application, but as the intervention is likely to change or disappear over the course of the years; also make sure the intervention is archived (Internet Archive, webcitation.org, and/or publishing the source code or screenshots/videos alongside the article). As pages behind login screens cannot be archived, consider creating demo pages which are accessible without login.

	1	2	3	4	5	
subitem not at all important	○	○	○	○	○	essential

Does your paper address subitem 5-vi?

Copy and paste relevant sections from the manuscript (include quotes in quotation marks "like this" to indicate direct quotes from your manuscript), or elaborate on this item by providing additional information not in the ms, or briefly explain why the item is not applicable/relevant for your study

this does not apply to this study

5-vii) Access

Access: Describe how participants accessed the application, in what setting/context, if they had to pay (or were paid) or not, whether they had to be a member of specific group. If known, describe how participants obtained “access to the platform and Internet” [1]. To ensure access for editors/reviewers/readers, consider to provide a “backdoor” login account or demo mode for reviewers/readers to explore the application (also important for archiving purposes, see vi).

	1	2	3	4	5	
subitem not at all important	○	○	○	○	◉	essential

Clear selection

Does your paper address subitem 5-vii? *

Copy and paste relevant sections from the manuscript (include quotes in quotation marks "like this" to indicate direct quotes from your manuscript), or elaborate on this item by providing additional information not in the ms, or briefly explain why the item is not applicable/relevant for your study

After consenting, patients were then registered on the SHERPA mHealth platform, but were only eligible to complete enrollment if a supporter agreed to participate as well. If a supporter was not eventually enrolled as well, the patient still received the patient text-messaging program, however they were excluded from further participation in the study. Randomization to pamphlet or mHealth augmented social support occurred after supporter enrollment.

Supporters were enrolled in the FANS program either in the ED during the initial contact with the patient or at a later time remotely by telephone. Enrollment consisted of verbally consenting the support person, confirming age>18, and confirming their ability to send and receive text messages. Randomization group was assigned by sealed envelope allocation after supporter consent to participate; the randomization sequence was generated by the senior study biostatistician. Supporters then completed baseline survey instruments and were registered on the SHERPA platform if randomized to the intervention arm, or received a pamphlet if randomized to the active control arm.

5-viii) Mode of delivery, features/functionalities/components of the intervention and comparator, and the theoretical framework

Describe mode of delivery, features/functionalities/components of the intervention and comparator, and the theoretical framework [6] used to design them (instructional strategy [1], behaviour change techniques, persuasive features, etc., see e.g., [7, 8] for terminology). This includes an in-depth description of the content (including where it is coming from and who developed it) [1],” whether [and how] it is tailored to individual circumstances and allows users to track their progress and receive feedback” [6]. This also includes a description of communication delivery channels and – if computer-mediated communication is a component – whether communication was synchronous or asynchronous [6]. It also includes information on presentation strategies [1], including page design principles, average amount of text on pages, presence of hyperlinks to other resources, etc. [1].

	1	2	3	4	5	
subitem not at all important	○	○	○	○	◉	essential

Clear selection

Does your paper address subitem 5-viii? *

Copy and paste relevant sections from the manuscript (include quotes in quotation marks "like this" to indicate direct quotes from your manuscript), or elaborate on this item by providing additional information not in the ms, or briefly explain why the item is not applicable/relevant for your study

The FANS messages focused on: 1) Instrumental support (tangible goods and actions), 2) Informational support (knowledge) and 3) Emotional support.14 Given the financial constraints the patients and family members of this population, FANS messages emphasized non-financial forms of instrumental support.

5-ix) Describe use parameters

Describe use parameters (e.g., intended “doses” and optimal timing for use). Clarify what instructions or recommendations were given to the user, e.g., regarding timing, frequency, heaviness of use, if any, or was the intervention used ad libitum.

	1	2	3	4	5	
subitem not at all important	○	○	○	○	◉	essential

Clear selection

Does your paper address subitem 5-ix?

Copy and paste relevant sections from the manuscript (include quotes in quotation marks "like this" to indicate direct quotes from your manuscript), or elaborate on this item by providing additional information not in the ms, or briefly explain why the item is not applicable/relevant for your study

"The development of the FANS support curriculum is described in a separate manuscript.13 In brief, the messages were developed from National Diabetes Education Program and American Diabetes Association (ADA) recommendations, synchronizing in content and time to 2 of the 3 daily patient messages."

5-x) Clarify the level of human involvement

Clarify the level of human involvement (care providers or health professionals, also technical assistance) in the e-intervention or as co-intervention (detail number and expertise of professionals involved, if any, as well as "type of assistance offered, the timing and frequency of the support, how it is initiated, and the medium by which the assistance is delivered". It may be necessary to distinguish between the level of human involvement required for the trial, and the level of human involvement required for a routine application outside of a RCT setting (discuss under item 21 – generalizability).

	1	2	3	4	5	
subitem not at all important	○	○	◉	○	○	essential

Clear selection

Does your paper address subitem 5-x?

Copy and paste relevant sections from the manuscript (include quotes in quotation marks "like this" to indicate direct quotes from your manuscript), or elaborate on this item by providing additional information not in the ms, or briefly explain why the item is not applicable/relevant for your study

this does not apply to this study

5-xi) Report any prompts/reminders used

Report any prompts/reminders used: Clarify if there were prompts (letters, emails, phone calls, SMS) to use the application, what triggered them, frequency etc. It may be necessary to distinguish between the level of prompts/reminders required for the trial, and the level of prompts/reminders for a routine application outside of a RCT setting (discuss under item 21 – generalizability).

	1	2	3	4	5	
subitem not at all important	○	○	○	○	●	essential

Clear selection

Does your paper address subitem 5-xi? *

Copy and paste relevant sections from the manuscript (include quotes in quotation marks "like this" to indicate direct quotes from your manuscript), or elaborate on this item by providing additional information not in the ms, or briefly explain why the item is not applicable/relevant for your study

Yes, daily text messages were sent, which is described throughout the study

5-xii) Describe any co-interventions (incl. training/support)

Describe any co-interventions (incl. training/support): Clearly state any interventions that are provided in addition to the targeted eHealth intervention, as ehealth intervention may not be designed as stand-alone intervention. This includes training sessions and support [1]. It may be necessary to distinguish between the level of training required for the trial, and the level of training for a routine application outside of a RCT setting (discuss under item 21 – generalizability.

	1	2	3	4	5	
subitem not at all important	○	○	●	○	○	essential

Clear selection

Does your paper address subitem 5-xii? *

Copy and paste relevant sections from the manuscript (include quotes in quotation marks "like this" to indicate direct quotes from your manuscript), or elaborate on this item by providing additional information not in the ms, or briefly explain why the item is not applicable/relevant for your study

No additional training happend or was mentioned

6a) Completely defined pre-specified primary and secondary outcome measures, including how and when they were assessed

Does your paper address CONSORT subitem 6a? *

Copy and paste relevant sections from the manuscript (include quotes in quotation marks "like this" to indicate direct quotes from your manuscript), or elaborate on this item by providing additional information not in the ms, or briefly explain why the item is not applicable/relevant for your study

Primary Outcome: The primary outcome was the change in A1C from baseline to six-months.
Secondary Outcomes: Secondary outcomes were:
1) 6-12 month post-intervention change in A1C
2) Baseline to 6 month change in BMI and Blood Pressure
3) Baseline to 6 month change in diabetes self-management behaviors (measure by SDSCA)

6a-i) Online questionnaires: describe if they were validated for online use and apply CHERRIES items to describe how the questionnaires were designed/deployed

If outcomes were obtained through online questionnaires, describe if they were validated for online use and apply CHERRIES items to describe how the questionnaires were designed/deployed [9].

	1	2	3	4	5	
subitem not at all important	○	○	○	○	○	essential

Does your paper address subitem 6a-i?

Copy and paste relevant sections from manuscript text

not used in this study, no online surveys

6a-ii) Describe whether and how “use” (including intensity of use/dosage) was defined/measured/monitored

Describe whether and how “use” (including intensity of use/dosage) was defined/measured/monitored (logins, logfile analysis, etc.). Use/adoption metrics are important process outcomes that should be reported in any ehealth trial.

	1	2	3	4	5	
subitem not at all important	○	○	○	○	◉	essential

Clear selection

Does your paper address subitem 6a-ii?

Copy and paste relevant sections from manuscript text

A sensitivity analysis confined to adherent participants (those who have not opted out of messages and have received 75% or greater of messages confirmed by message delivery platform) was planned but was not possible due to limitations in the cellular service providers most patients had.

6a-iii) Describe whether, how, and when qualitative feedback from participants was obtained

Describe whether, how, and when qualitative feedback from participants was obtained (e.g., through emails, feedback forms, interviews, focus groups).

	1	2	3	4	5	
subitem not at all important	○	○	○	○	◉	essential

Clear selection

Does your paper address subitem 6a-iii?

Copy and paste relevant sections from manuscript text

this is part of a separate study

6b) Any changes to trial outcomes after the trial commenced, with reasons

Does your paper address CONSORT subitem 6b? *

Copy and paste relevant sections from the manuscript (include quotes in quotation marks "like this" to indicate direct quotes from your manuscript), or elaborate on this item by providing additional information not in the ms, or briefly explain why the item is not applicable/relevant for your study

no changes were made to the outcomes

7a) How sample size was determined

NPT: When applicable, details of whether and how the clustering by care provides or centers was addressed

7a-i) Describe whether and how expected attrition was taken into account when calculating the sample size

Describe whether and how expected attrition was taken into account when calculating the sample size.

	1	2	3	4	5	
subitem not at all important	○	○	○	○	◉	essential

Clear selection

Does your paper address subitem 7a-i?

Copy and paste relevant sections from manuscript title (include quotes in quotation marks "like this" to indicate direct quotes from your manuscript), or elaborate on this item by providing additional information not in the ms, or briefly explain why the item is not applicable/relevant for your study

"Sample size and effect size calculations
We planned to enroll 166 patient-supporter dyads, assuming a 30% loss to follow up,29 to yield a planned sample size of 116 total dyads. Power of 0.80 and two-sided alpha of 0.05, using the standard deviation of final A1C of 1.6 (the value of our prior trials), provided the ability to detect a mean difference in change of A1C of 0.84 between the two groups at six month follow up.30"

7b) When applicable, explanation of any interim analyses and stopping guidelines

Does your paper address CONSORT subitem 7b? *

Copy and paste relevant sections from the manuscript (include quotes in quotation marks "like this" to indicate direct quotes from your manuscript), or elaborate on this item by providing additional information not in the ms, or briefly explain why the item is not applicable/relevant for your study

No interim analysis or stopping guidelines were established

8a) Method used to generate the random allocation sequence

NPT: When applicable, how care providers were allocated to each trial group

Does your paper address CONSORT subitem 8a? *

Copy and paste relevant sections from the manuscript (include quotes in quotation marks "like this" to indicate direct quotes from your manuscript), or elaborate on this item by providing additional information not in the ms, or briefly explain why the item is not applicable/relevant for your study

". Randomization group was assigned by sealed envelope allocation after supporter consent to participate; the randomization sequence was generated by the senior study biostatistician. "

8b) Type of randomisation; details of any restriction (such as blocking and block size)

Does your paper address CONSORT subitem 8b? *

Copy and paste relevant sections from the manuscript (include quotes in quotation marks "like this" to indicate direct quotes from your manuscript), or elaborate on this item by providing additional information not in the ms, or briefly explain why the item is not applicable/relevant for your study

". Randomization group was assigned by sealed envelope allocation after supporter consent to participate; the randomization sequence was generated by the senior study biostatistician. "

9) Mechanism used to implement the random allocation sequence (such as sequentially numbered containers), describing any steps taken to conceal the sequence until interventions were assigned

Does your paper address CONSORT subitem 9? *

Copy and paste relevant sections from the manuscript (include quotes in quotation marks "like this" to indicate direct quotes from your manuscript), or elaborate on this item by providing additional information not in the ms, or briefly explain why the item is not applicable/relevant for your study

". Randomization group was assigned by sealed envelope allocation after supporter consent to participate; the randomization sequence was generated by the senior study biostatistician. "

10) Who generated the random allocation sequence, who enrolled participants, and who assigned participants to interventions

Does your paper address CONSORT subitem 10? *

Copy and paste relevant sections from the manuscript (include quotes in quotation marks "like this" to indicate direct quotes from your manuscript), or elaborate on this item by providing additional information not in the ms, or briefly explain why the item is not applicable/relevant for your study

". Randomization group was assigned by sealed envelope allocation after supporter consent to participate; the randomization sequence was generated by the senior study biostatistician. "

11a) If done, who was blinded after assignment to interventions (for example, participants, care providers, those assessing outcomes) and how

NPT: Whether or not administering co-interventions were blinded to group assignment

11a-i) Specify who was blinded, and who wasn't

Specify who was blinded, and who wasn't. Usually, in web-based trials it is not possible to blind the participants [1, 3] (this should be clearly acknowledged), but it may be possible to blind outcome assessors, those doing data analysis or those administering co-interventions (if any).

	1	2	3	4	5	
subitem not at all important	○	○	○	◉	○	essential

Clear selection

Does your paper address subitem 11a-i? *

Copy and paste relevant sections from the manuscript (include quotes in quotation marks "like this" to indicate direct quotes from your manuscript), or elaborate on this item by providing additional information not in the ms, or briefly explain why the item is not applicable/relevant for your study

"This study was an unblinded, randomized, parallel, active controlled intervention with 1:1 allocation ratio. "

11a-ii) Discuss e.g., whether participants knew which intervention was the "intervention of interest" and which one was the "comparator"

Informed consent procedures (4a-ii) can create biases and certain expectations - discuss e.g., whether participants knew which intervention was the "intervention of interest" and which one was the "comparator".

	1	2	3	4	5	
subitem not at all important	○	○	○	○	◉	essential

Clear selection

Does your paper address subitem 11a-ii?

Copy and paste relevant sections from the manuscript (include quotes in quotation marks "like this" to indicate direct quotes from your manuscript), or elaborate on this item by providing additional information not in the ms, or briefly explain why the item is not applicable/relevant for your study

"This study was an unblinded, randomized, parallel, active controlled intervention with 1:1 allocation ratio."

11b) If relevant, description of the similarity of interventions

(this item is usually not relevant for ehealth trials as it refers to similarity of a placebo or sham intervention to a active medication/intervention)

Does your paper address CONSORT subitem 11b? *

Copy and paste relevant sections from the manuscript (include quotes in quotation marks "like this" to indicate direct quotes from your manuscript), or elaborate on this item by providing additional information not in the ms, or briefly explain why the item is not applicable/relevant for your study

"All subjects received a SMS text-message based mHealth curriculum for diabetes self-management. At the patient level, supporters were randomized to receive a supporter curriculum delivered by SMS text-message or by pamphlet organized by week of the curriculum."

12a) Statistical methods used to compare groups for primary and secondary outcomes

NPT: When applicable, details of whether and how the clustering by care providers or centers was addressed

Does your paper address CONSORT subitem 12a? *

Copy and paste relevant sections from the manuscript (include quotes in quotation marks "like this" to indicate direct quotes from your manuscript), or elaborate on this item by providing additional information not in the ms, or briefly explain why the item is not applicable/relevant for your study

"Primary Outcome Analysis:
The primary outcome was the change in A1C from baseline to six-months. Participants who completed the twelve-month study provided two outcome measures of six-month change: a zero-six month measure of treatment efficacy, and a six-twelve month measure of sustainability of treatment effect. Normality of the outcome variable (six-month change) was graphically evaluated. We employed a mixed effects linear regression model to account for correlated outcome data (zero-six month and six-twelve month changes) and loss to follow up. Analyses were conducted by intent-to-treat, with participants analyzed according to their randomized intervention regardless of adherence. The linear mixed effects model included a random intercept term for participants. Fixed effects included treatment allocation, initial level of A1C (zero month measure for treatment efficacy, six-month measure for sustainability), and a covariate of study period (zero-six month, six-twelve month). The main effect of treatment tested for group differences over both zero-six and six-twelve month periods. An interaction term of treatment by study period tested for differences in treatment effects by study period; treatment effects were estimated and tested for differences by study period in this interaction model. Model assumptions including normality of model residuals and homogeneity of variance were evaluated.

We examined for confounding by the change in intervention efficacy estimate method, with a cut off of 20% change. Potential confounders examined were gender of patient, gender of supporter, patient age, race and ethnic group, language preference, health literacy, patient and supporter technologic capacity, patient and supporter living at the same address, baseline family supportive behaviors and supporter being immediately present for enrollment versus requiring multiple contact attempts.

After the regression model was built, predicted mean differences between the groups in A1C at 6 months were examined with the margins and contrast postestimation tools in Stata at the 6-month timepoint. A sensitivity analysis confined to adherent participants (those who have not opted out of messages and have received 75% or greater of messages confirmed by message delivery platform) was planned but was not possible due to limitations in the cellular service providers most patients had.

Secondary Outcomes Analysis:
Secondary outcomes of 6-12 month intervention efficacy of TExT-MED+FANS vs TExT-MED+pamphlet social support on A1C and 0-6 month intervention efficacy of TExT-MED+FANS vs TExT-MED+ pamphlet social support on Clinical Outcomes and Diabetes Self-Care Behaviors were examined with the same mixed effects models as the primary outcome."

12a-i) Imputation techniques to deal with attrition / missing values

Imputation techniques to deal with attrition / missing values: Not all participants will use the intervention/comparator as intended and attrition is typically high in ehealth trials. Specify how participants who did not use the application or dropped out from the trial were treated in the statistical analysis (a complete case analysis is strongly discouraged, and simple imputation techniques such as LOCF may also be problematic [4]).

	1	2	3	4	5	
subitem not at all important	○	○	○	○	◉	essential

Clear selection

Does your paper address subitem 12a-i? *

Copy and paste relevant sections from the manuscript (include quotes in quotation marks "like this" to indicate direct quotes from your manuscript), or elaborate on this item by providing additional information not in the ms, or briefly explain why the item is not applicable/relevant for your study

this did not occur in this study

12b) Methods for additional analyses, such as subgroup analyses and adjusted analyses

Does your paper address CONSORT subitem 12b? *

Copy and paste relevant sections from the manuscript (include quotes in quotation marks "like this" to indicate direct quotes from your manuscript), or elaborate on this item by providing additional information not in the ms, or briefly explain why the item is not applicable/relevant for your study

"A Priori Subgroup Analysis
To determine if subgroups of participants are differentially affected by the intervention, secondary analyses evaluating intervention moderators were planned a priori. For A1C and each of the secondary outcomes, interaction terms (randomized intervention-by-moderator product terms) were added to the mixed effects linear models described above. Variables evaluated as moderators included patient and supporter gender, race and ethnicity in 4 categories, language preference., health literacy (low health literacy defined as Brief Health Literacy Score of less than 12), new diagnosis of diabetes (less than 12 months), baseline frequency of mobile usage as high or low based on latent profiles previously described,28 and physically proximity to supporter. Intervention effects were estimated by levels of the moderator for significant moderators only at a p value of 0.05 for the interaction term between randomization group and moderator.

Post Hoc Subgroup Analysis
During enrollment, substantial differences in baseline support and contact between patients and their selected supporters became evident, as some supporters took up to three weeks to complete enrollment procedures. We conducted a subgroup analysis based on supporter immediate availability for enrollment versus delayed enrollment. "

X26) REB/IRB Approval and Ethical Considerations [recommended as subheading under "Methods"] (not a CONSORT item)

X26-i) Comment on ethics committee approval

	1	2	3	4	5	
subitem not at all important	○	○	○	○	◉	essential

Clear selection

Does your paper address subitem X26-i?

Copy and paste relevant sections from the manuscript (include quotes in quotation marks "like this" to indicate direct quotes from your manuscript), or elaborate on this item by providing additional information not in the ms, or briefly explain why the item is not applicable/relevant for your study

"IRB approval for this study was obtained prior to study initiation from the USC Health Sciences Institutional Review Board (HS-17-00406)."

x26-ii) Outline informed consent procedures

Outline informed consent procedures e.g., if consent was obtained offline or online (how? Checkbox, etc.?), and what information was provided (see 4a-ii). See [6] for some items to be included in informed consent documents.

	1	2	3	4	5	
subitem not at all important	○	○	○	●	○	essential

Clear selection

Does your paper address subitem X26-ii?

Copy and paste relevant sections from the manuscript (include quotes in quotation marks "like this" to indicate direct quotes from your manuscript), or elaborate on this item by providing additional information not in the ms, or briefly explain why the item is not applicable/relevant for your study

"Research assistants (RAs) explained the purpose of the study and obtained consent in the ED. Patients were aware at enrollment that the designated supporter could receive multiple text-messages per day and would be prompted to offer increased support."

X26-iii) Safety and security procedures

Safety and security procedures, incl. privacy considerations, and any steps taken to reduce the likelihood or detection of harm (e.g., education and training, availability of a hotline)

	1	2	3	4	5	
subitem not at all important	○	○	○	○	◉	essential

Clear selection

Does your paper address subitem X26-iii?

Copy and paste relevant sections from the manuscript (include quotes in quotation marks "like this" to indicate direct quotes from your manuscript), or elaborate on this item by providing additional information not in the ms, or briefly explain why the item is not applicable/relevant for your study

"Safety Monitoring
There was a potential risk of hypoglycemia as patients improved their medication adherence if patients were previously prescribed insulin or oral insulin secretagogues. Patient knowledge of symptoms and treatment for hypoglycemia is low; hypoglycemia was the first focus of educational messages sent to both supporters and patients. On enrollment, patients were instructed to report episodes of hypoglycemia to the research team and to call their primary care team. If the patient did not have a regular primary care source, the research team instructed the patient to visit the urgent access center at LAC+USC. We evaluated for a difference in patient reported hypoglycemic events between the two groups at six and twelve months."

RESULTS

13a) For each group, the numbers of participants who were randomly assigned, received intended treatment, and were analysed for the primary outcome

NPT: The number of care providers or centers performing the intervention in each group and the number of patients treated by each care provider in each center

Does your paper address CONSORT subitem 13a? *

Copy and paste relevant sections from the manuscript (include quotes in quotation marks "like this" to indicate direct quotes from your manuscript), or elaborate on this item by providing additional information not in the ms, or briefly explain why the item is not applicable/relevant for your study

"Study follow-up
We obtained measures of our primary outcome, A1C at 6 months, in 52% of the FANS intervention group, and 60% of the pamphlet support control group. In the FANS intervention group, 8 patients dropped out of the study, and 27 were lost to follow up. In the pamphlet support group, 5 patients dropped out of the study and 29 were lost to follow up. At 12 months, after a maintenance phase with no text-messages, we obtained A1C measurements for 50 patients (58%) in the FANS group, and 56 of the pamphlet support group (65%). In the FANS group, 7 patients withdrew from the study during the maintenance phase, but 5 patients followed up who were not available at 6 months. In the pamphlet support group, 5 patients withdrew from the study during the maintenance phase, but 4 more followed up who were not available at 6 months. "

13b) For each group, losses and exclusions after randomisation, together with reasons

Does your paper address CONSORT subitem 13b? (NOTE: Preferably, this is shown in a CONSORT flow diagram) *

Copy and paste relevant sections from the manuscript (include quotes in quotation marks "like this" to indicate direct quotes from your manuscript), or elaborate on this item by providing additional information not in the ms, or briefly explain why the item is not applicable/relevant for your study

"Study follow-up
We obtained measures of our primary outcome, A1C at 6 months, in 52% of the FANS intervention group, and 60% of the pamphlet support control group. In the FANS intervention group, 8 patients dropped out of the study, and 27 were lost to follow up. In the pamphlet support group, 5 patients dropped out of the study and 29 were lost to follow up. At 12 months, after a maintenance phase with no text-messages, we obtained A1C measurements for 50 patients (58%) in the FANS group, and 56 of the pamphlet support group (65%). In the FANS group, 7 patients withdrew from the study during the maintenance phase, but 5 patients followed up who were not available at 6 months. In the pamphlet support group, 5 patients withdrew from the study during the maintenance phase, but 4 more followed up who were not available at 6 months. "

13b-i) Attrition diagram

Strongly recommended: An attrition diagram (e.g., proportion of participants still logging in or using the intervention/comparator in each group plotted over time, similar to a survival curve) or other figures or tables demonstrating usage/dose/engagement.

	1	2	3	4	5	
subitem not at all important	○	○	○	○	●	essential

Clear selection

Does your paper address subitem 13b-i?

Copy and paste relevant sections from the manuscript or cite the figure number if applicable (include quotes in quotation marks "like this" to indicate direct quotes from your manuscript), or elaborate on this item by providing additional information not in the ms, or briefly explain why the item is not applicable/relevant for your study

figure is in study manuscript file

14a) Dates defining the periods of recruitment and follow-up

Does your paper address CONSORT subitem 14a? *

Copy and paste relevant sections from the manuscript (include quotes in quotation marks "like this" to indicate direct quotes from your manuscript), or elaborate on this item by providing additional information not in the ms, or briefly explain why the item is not applicable/relevant for your study

Patients with diabetes were screened and enrolled by surveying the ED electronic patient tracking system from July 2017 to October 2018.

14a-i) Indicate if critical "secular events" fell into the study period

Indicate if critical "secular events" fell into the study period, e.g., significant changes in Internet resources available or "changes in computer hardware or Internet delivery resources"

	1	2	3	4	5	
subitem not at all important	○	○	○	○	◉	essential

Clear selection

Does your paper address subitem 14a-i?

Copy and paste relevant sections from the manuscript (include quotes in quotation marks "like this" to indicate direct quotes from your manuscript), or elaborate on this item by providing additional information not in the ms, or briefly explain why the item is not applicable/relevant for your study

the trial concluded just prior to the COVID pandemic, and thus does not impact the results

14b) Why the trial ended or was stopped (early)

Does your paper address CONSORT subitem 14b? *

Copy and paste relevant sections from the manuscript (include quotes in quotation marks "like this" to indicate direct quotes from your manuscript), or elaborate on this item by providing additional information not in the ms, or briefly explain why the item is not applicable/relevant for your study

trial concluded at normal time, it did not stop early

15) A table showing baseline demographic and clinical characteristics for each group

NPT: When applicable, a description of care providers (case volume, qualification, expertise, etc.) and centers (volume) in each group

Does your paper address CONSORT subitem 15? *

Copy and paste relevant sections from the manuscript (include quotes in quotation marks "like this" to indicate direct quotes from your manuscript), or elaborate on this item by providing additional information not in the ms, or briefly explain why the item is not applicable/relevant for your study

this is included in teh manuscript file

15-i) Report demographics associated with digital divide issues

In ehealth trials it is particularly important to report demographics associated with digital divide issues, such as age, education, gender, social-economic status, computer/Internet/ehealth literacy of the participants, if known.

	1	2	3	4	5	
subitem not at all important	○	○	○	○	◉	essential

Clear selection

Does your paper address subitem 15-i? *

Copy and paste relevant sections from the manuscript (include quotes in quotation marks "like this" to indicate direct quotes from your manuscript), or elaborate on this item by providing additional information not in the ms, or briefly explain why the item is not applicable/relevant for your study

this is reported in an another study manuscript

16) For each group, number of participants (denominator) included in each analysis and whether the analysis was by original assigned groups

16-i) Report multiple “denominators” and provide definitions

Report multiple “denominators” and provide definitions: Report N’s (and effect sizes) “across a range of study participation [and use] thresholds” [1], e.g., N exposed, N consented, N used more than x times, N used more than y weeks, N participants “used” the intervention/comparator at specific pre-defined time points of interest (in absolute and relative numbers per group). Always clearly define “use” of the intervention.

	1	2	3	4	5	
subitem not at all important	○	○	○	○	◉	essential

Clear selection

Does your paper address subitem 16-i? *

Copy and paste relevant sections from the manuscript (include quotes in quotation marks "like this" to indicate direct quotes from your manuscript), or elaborate on this item by providing additional information not in the ms, or briefly explain why the item is not applicable/relevant for your study

only one N was used for the analysis

16-ii) Primary analysis should be intent-to-treat

Primary analysis should be intent-to-treat, secondary analyses could include comparing only “users”, with the appropriate caveats that this is no longer a randomized sample (see 18-i).

	1	2	3	4	5	
subitem not at all important	○	○	○	○	◉	essential

Clear selection

Does your paper address subitem 16-ii?

Copy and paste relevant sections from the manuscript (include quotes in quotation marks "like this" to indicate direct quotes from your manuscript), or elaborate on this item by providing additional information not in the ms, or briefly explain why the item is not applicable/relevant for your study

"Analyses were conducted by intent-to-treat, with participants analyzed according to their randomized intervention regardless of adherence. "

17a) For each primary and secondary outcome, results for each group, and the estimated effect size and its precision (such as 95% confidence interval)

Does your paper address CONSORT subitem 17a? *

Copy and paste relevant sections from the manuscript (include quotes in quotation marks "like this" to indicate direct quotes from your manuscript), or elaborate on this item by providing additional information not in the ms, or briefly explain why the item is not applicable/relevant for your study

these are all represented in the results tables

17a-i) Presentation of process outcomes such as metrics of use and intensity of use

In addition to primary/secondary (clinical) outcomes, the presentation of process outcomes such as metrics of use and intensity of use (dose, exposure) and their operational definitions is critical. This does not only refer to metrics of attrition (13-b) (often a binary variable), but also to more continuous exposure metrics such as “average session length”. These must be accompanied by a technical description how a metric like a “session” is defined (e.g., timeout after idle time) [1] (report under item 6a).

	1	2	3	4	5	
subitem not at all important	○	○	○	○	○	essential

Does your paper address subitem 17a-i?

Copy and paste relevant sections from the manuscript (include quotes in quotation marks "like this" to indicate direct quotes from your manuscript), or elaborate on this item by providing additional information not in the ms, or briefly explain why the item is not applicable/relevant for your study

this is not relevant to the study, as it was a push intervention

17b) For binary outcomes, presentation of both absolute and relative effect sizes is recommended

Does your paper address CONSORT subitem 17b? *

Copy and paste relevant sections from the manuscript (include quotes in quotation marks "like this" to indicate direct quotes from your manuscript), or elaborate on this item by providing additional information not in the ms, or briefly explain why the item is not applicable/relevant for your study

this is not relevant to this study

18) Results of any other analyses performed, including subgroup analyses and adjusted analyses, distinguishing pre-specified from exploratory

Does your paper address CONSORT subitem 18? *

Copy and paste relevant sections from the manuscript (include quotes in quotation marks "like this" to indicate direct quotes from your manuscript), or elaborate on this item by providing additional information not in the ms, or briefly explain why the item is not applicable/relevant for your study

"Subgroup Analyses of Primary Outcome: 0-6 month efficacy of TExT-MED+FANS vs TExT-MED+pamphlet social support on A1C
Among patients with a new diagnosis of diabetes, FANS group patients improved their A1C to a greater degree than patients in the pamphlet social support arm, mean between group difference of 1.96%mg/dL(95%CI -3.81 to -0.125, pbetween group= 0.036). We found no differences in intervention effects at 0-6 months based on subgroups of gender, race and ethnicity, language preference, health literacy, baseline frequency of mobile usage, physical proximity to supporters, or baseline social support.

Subgroup Analyses of Secondary Outcome: 6-12 month efficacy of maintenance of TExT-MED+FANS vs TExT-MED+pamphlet social support on A1C
In the same prespecified subgroup analysis as the 6 month timeframe, we found that among patients with a new diagnosis of diabetes, FANS group patients improved their A1C to a greater degree than patients in the pamphlet social support arm, with a predicted between group difference of 2.40%mg/dL (95%CI -4.33 to -0.47, pbetween group= 0.002) at 12 months. We also found that patients who preferred texts in English maintained better glycemic control with the FANS intervention compared to the pamphlet augmented social support, with a predicted between group difference of 2.53%mg/dL(95%CI -4.15 to -0.91, pbetween group= 0.015) at 12 months. We found no differences in A1C at 6-12 months based on gender, race and ethnicity, health literacy, baseline frequency of mobile usage, physical proximity to supporters, and baseline social support. "

18-i) Subgroup analysis of comparing only users

A subgroup analysis of comparing only users is not uncommon in ehealth trials, but if done, it must be stressed that this is a self-selected sample and no longer an unbiased sample from a randomized trial (see 16-iii).

	1	2	3	4	5	
subitem not at all important	○	○	○	○	○	essential

Does your paper address subitem 18-i?

Copy and paste relevant sections from the manuscript (include quotes in quotation marks "like this" to indicate direct quotes from your manuscript), or elaborate on this item by providing additional information not in the ms, or briefly explain why the item is not applicable/relevant for your study

this did not occur in this study

19) All important harms or unintended effects in each group

(for specific guidance see CONSORT for harms)

Does your paper address CONSORT subitem 19? *

Copy and paste relevant sections from the manuscript (include quotes in quotation marks "like this" to indicate direct quotes from your manuscript), or elaborate on this item by providing additional information not in the ms, or briefly explain why the item is not applicable/relevant for your study

"Safety
The study group had a low adverse event profile. One patient's death due to urosepsis in the FANS arm during the post-intervention maintenance phase was determined to be unrelated to the intervention after review by the local institutional review board. Severe hypoglycemic events (blood glucose <70 mg/dl) at 6 months were self-reported in 20% of FANS arm patients reporting at least one episode during the intervention phase, and 37% in the pamphlet support arm reporting at least one hypoglycemic event in the intervention phase(p=0.051). "

19-i) Include privacy breaches, technical problems

Include privacy breaches, technical problems. This does not only include physical "harm" to participants, but also incidents such as perceived or real privacy breaches [1], technical problems, and other unexpected/unintended incidents. "Unintended effects" also includes unintended positive effects [2].

	1	2	3	4	5	
subitem not at all important	○	○	○	○	○	essential

Does your paper address subitem 19-i?

Copy and paste relevant sections from the manuscript (include quotes in quotation marks "like this" to indicate direct quotes from your manuscript), or elaborate on this item by providing additional information not in the ms, or briefly explain why the item is not applicable/relevant for your study

did not occur in this study

19-ii) Include qualitative feedback from participants or observations from staff/researchers

Include qualitative feedback from participants or observations from staff/researchers, if available, on strengths and shortcomings of the application, especially if they point to unintended/unexpected effects or uses. This includes (if available) reasons for why people did or did not use the application as intended by the developers.

	1	2	3	4	5	
subitem not at all important	○	○	○	○	○	essential

Does your paper address subitem 19-ii?

Copy and paste relevant sections from the manuscript (include quotes in quotation marks "like this" to indicate direct quotes from your manuscript), or elaborate on this item by providing additional information not in the ms, or briefly explain why the item is not applicable/relevant for your study

this is presented in another study manuscript

DISCUSSION

22) Interpretation consistent with results, balancing benefits and harms, and considering other relevant evidence

NPT: In addition, take into account the choice of the comparator, lack of or partial blinding, and unequal expertise of care providers or centers in each group

22-i) Restate study questions and summarize the answers suggested by the data, starting with primary outcomes and process outcomes (use)

Restate study questions and summarize the answers suggested by the data, starting with primary outcomes and process outcomes (use).

	1	2	3	4	5	
subitem not at all important	○	○	○	○	○	essential

Does your paper address subitem 22-i? *

Copy and paste relevant sections from the manuscript (include quotes in quotation marks "like this" to indicate direct quotes from your manuscript), or elaborate on this item by providing additional information not in the ms, or briefly explain why the item is not applicable/relevant for your study

"Discussion:

Principal Results

To test if augmenting existing social support via mHealth for safety-net ED patients improved diabetes self-management behaviors and glycemic control compared to a pamphlet-based support intervention, we conducted the randomized TExT-MED+FANS study. We designed a randomized trial with some pragmatic features to increase potential translation to clinical care: a focus on enrollment of patients while in the emergency department and not requiring their identified supporter to also be physically present. The study population was recruited from a medically vulnerable group, with low access to medical homes,12 and had a high potential to benefit from both the patient focused and social support interventions. While all patients improved their glycemic control and self-management behaviors to a clinically significant degree, we did not find that the FANS curriculum sent via text-message to patient identified supporters provided additional benefit over a pamphlet-based support curriculum sent to supporters.

Comparison to Prior Literature

We found similar improvement of glycemic control, self-management behaviors, psychological outcomes, and social support measures between the FANS-supported group and pamphlet social support group. Prior pooled analysis of mHealth interventions to improve diabetes self-management have shown mean A1C improvements of 0.5% across types of diabetes and mHealth modalities31, while a prior meta-analysis for nine traditional social support interventions for diabetes self-management showed an improvement in A1C [illegible]

of 0.25% at 3 months (95% CI −0.40 to -0.11)32. There is not sufficient literature to generate pooled estimates of mHealth/eHealth-based social support for diabetes self-management 33. As there was no inactive control group in this trial, the potential benefit of TExT-MED plus social support augmentation via mHealth may be subject to a floor effect given the TExT-MED plus social support pamphlet provided. In this study, we found the overall combined sample A1C improvement of 1.42% at 6 months (95% CI 0.82 to 2.02) and the maintenance of that improvement at 12 months with a washout period change of 0.06% (95%CI -0.47 to 0.34.) The larger improvement in A1c found in the study compare with the literature is encouraging, suggesting addition of social supporter to an mHealth diabetes program, either via mHealth texting or pamphlet, has the potential to improve the long-term health outcomes of safety-net ED patients. Additionally, we found the intervention group reported few instances of hypoglycemia, an important safety outcome for this intervention. The social support mHealth texting to augment support for patients has high potential to be translated to a system wide intervention, given the possibility of remote self-enrollment by patients and automated delivery.

Patients who benefitted the most from the mHealth augmented social support of the FANS curriculum to supporters compared to the pamphlet augmented support were those who were diagnosed with diabetes in the year prior to enrollment. Diabetes and other chronic medical conditions require complex lifestyle changes for the patient and their family members. Patients and family members may be largely unfamiliar with best self-management practices .34-36 A Cochrane Review analysis on diabetes self-management education interventions versus standard care for patients with newly diagnosed diabetes showed mean differences in A1C (-0.21%, 95% CI -0.38 to -0.04) 12 months after initial intervention of educational materials.37 National and international guidelines recommend that diabetes self-management education and support be provided to patients with diabetes upon their initial diagnosis and this benefit is covered by many medical insurers.38,39 However, less than 10% of patients receive this training internationally, with even lower rates in the US and in under-resourced environments.40-42 Additionally, there are disparities in barriers to attendance at these trainings by type of medical insurance, socioeconomic status, language, and mental health conditions.40,43,44 Educational interventions for those with newly diagnosed diabetes need to reach patients and their families at the critical time when their health behaviors require drastic change. mHealth-based training that incorporates family members and close friends, such as TExT-MED+FANS, can overcome some of these barriers.

Despite TExT-MED+FANS' development to specifically address the needs of a predominantly Spanish-speaking population at the LAC+USC Emergency Department, including careful translation of messages and extensive pre-testing for cultural and linguistic congruency,13 patients who preferred text-messages in English maintained their improvements in glycemic control to a greater degree. This disparity by language speaks to the continued obstacles that Spanish-speaking patients experience in accessing providers who are culturally competent and able to speak their preferred language, resulting in poor health outcomes. Spanish speakers in the United States are disproportionately burdened by T2DM than their English-speaking counterparts and face increased barriers to self-management support, especially when they have a non-language congruent primary healthcare provider.45-47

However, Spanish-speaking groups have benefited from mHealth interventions: the Spanish-speaking and medically underserved patients who enrolled in the Vida Health Diabetes Management Program (a novel, culturally adapted, Spanish-language mHealth diabetes program for glycemic control based out of continuity care clinics) showed an impressive decrease of -1.23% at 1-year post enrollment.48 The initial TExT-MED patient focused curriculum showed a 0.8% decrease among Spanish-speaking emergency department patients at 6 months after enrollment.9 The critical link in addressing language and cultural barriers to adequate continuity care for diabetes management may not be fully addressed in individual level interventions such as SMS text-message; integration into medical homes may be required to improve patient outcomes in linguistically underserved communities.

A relatively unique feature of this study in the mHealth-based social support for diabetes self-management literature is the requirement that all patients were required to select a family member or friend to be a supporter. Several trials of social support have shown improvements in diabetes behaviors and glycemic control when a patient elects to enroll a supporter or informal caregiver compared to patients who do not select a supporter.49-51 However, having higher baseline social support is generally associated with improved diabetes control and self-management behaviors in cross-sectional studies.52-54 One diabetes mHealth social support pilot intervention for physical activity in a similar population also restricted enrollment to patients who had an available supporter, and exhibited increased perceived social support for the social support arm, but no difference in physical activity recorded by a pedometer.55 By requiring a supporter for all patient participants, we can better understand how augmenting social support adds to patient-focused interventions, rather than measuring possible confounding by having sufficient baseline support to be able to identify a supporter. In our context, we found a comparable benefit between the mHealth augmented social support intervention and the pamphlet support comparison groups added to the patient focused curriculum. The method of including a social support person may only need a well-designed educational mailing on how to best support a loved one with diabetes. "

22-ii) Highlight unanswered new questions, suggest future research

Highlight unanswered new questions, suggest future research.

	1	2	3	4	5	
subitem not at all important	○	○	○	○	○	essential

Does your paper address subitem 22-ii?

Copy and paste relevant sections from the manuscript (include quotes in quotation marks "like this" to indicate direct quotes from your manuscript), or elaborate on this item by providing additional information not in the ms, or briefly explain why the item is not applicable/relevant for your study

"

A relatively unique feature of this study in the mHealth-based social support for diabetes self-management literature is the requirement that all patients were required to select a family member or friend to be a supporter. Several trials of social support have shown improvements in diabetes behaviors and glycemic control when a patient elects to enroll a supporter or informal caregiver compared to patients who do not select a supporter.49-51 However, having higher baseline social support is generally associated with improved diabetes control and self-management behaviors in cross-sectional studies.52-54 One diabetes mHealth social support pilot intervention for physical activity in a similar population also restricted enrollment to patients who had an available supporter, and exhibited increased perceived social support for the social support arm, but no difference in physical activity recorded by a pedometer.55 By requiring a supporter for all patient participants, we can better understand how augmenting social support adds to patient-focused interventions, rather than measuring possible confounding by having sufficient baseline support to be able to identify a supporter. In our context, we found a comparable benefit between the mHealth augmented social support intervention and the pamphlet support comparison groups added to the patient focused curriculum. The method of including a social support person may only need a well-designed educational mailing on how to best support a loved one with diabetes. "

20) Trial limitations, addressing sources of potential bias, imprecision, and, if relevant, multiplicity of analyses

20-i) Typical limitations in ehealth trials

Typical limitations in ehealth trials: Participants in ehealth trials are rarely blinded. Ehealth trials often look at a multiplicity of outcomes, increasing risk for a Type I error. Discuss biases due to non-use of the intervention/usability issues, biases through informed consent procedures, unexpected events.

	1	2	3	4	5	
subitem not at all important	○	○	○	○	○	essential

Does your paper address subitem 20-i? *

Copy and paste relevant sections from the manuscript (include quotes in quotation marks "like this" to indicate direct quotes from your manuscript), or elaborate on this item by providing additional information not in the ms, or briefly explain why the item is not applicable/relevant for your study

"Limitations:
Despite the importance of this study in this high need population, there are several limitations. First, there was no comparison group with no activation of supporters, making it difficult to determine what improvements were solely due to TExT-MED alone. Assessing "dose" of messaging was difficult, as there were no messages that bounced back, and participants used their own devices, limiting ability to determine how many messages were actually read. This decision to utilize participants' own phones and to utilize universally compatible SMS text-messaging was an important decision to maintain the pragmatic nature of this trial. Additionally, we did not include a social desirability measure in baseline data, which may impact the self-reported behavior measures. However, given that there was no inactive comparison group, this likely had limited impact. Health behaviors were measured by self-report given financial constraints of the study; remote pill monitoring, mobile-connected pedometers and extensive diet records were not possible and limit the pragmatic nature of the trial. The population of this study was constrained to ED patients with poor control of their diabetes and limits generalizability to primary care populations and patients with a more modest need for improvement in their diabetes management. A potentially serious limitation is the significant loss to follow up in this highly transient patient population. However, the patients who completed the trial were generally similar to those who did not, with the exception that patients who stayed in the study had slightly higher perceived attitudes that the social support they received was negative, potentially attenuating the estimated effect of the FANS curriculum."

21) Generalisability (external validity, applicability) of the trial findings

NPT: External validity of the trial findings according to the intervention, comparators, patients, and care providers or centers involved in the trial

21-i) Generalizability to other populations

Generalizability to other populations: In particular, discuss generalizability to a general Internet population, outside of a RCT setting, and general patient population, including applicability of the study results for other organizations

	1	2	3	4	5	
subitem not at all important	○	○	○	○	○	essential

Does your paper address subitem 21-i?

Copy and paste relevant sections from the manuscript (include quotes in quotation marks "like this" to indicate direct quotes from your manuscript), or elaborate on this item by providing additional information not in the ms, or briefly explain why the item is not applicable/relevant for your study

"Despite TExT-MED+FANS' development to specifically address the needs of a predominantly Spanish-speaking population at the LAC+USC Emergency Department, including careful translation of messages and extensive pre-testing for cultural and linguistic congruency,13 patients who preferred text-messages in English maintained their improvements in glycemic control to a greater degree. This disparity by language speaks to the continued obstacles that Spanish-speaking patients experience in accessing providers who are culturally competent and able to speak their preferred language, resulting in poor health outcomes. Spanish speakers in the United States are disproportionately burdened by T2DM than their English-speaking counterparts and face increased barriers to self-management support, especially when they have a non-language congruent primary healthcare provider.45-47 However, Spanish-speaking groups have benefited from mHealth interventions: the Spanish-speaking and medically underserved patients who enrolled in the Vida Health Diabetes Management Program (a novel, culturally adapted, Spanish-language mHealth diabetes program for glycemic control based out of continuity care clinics) showed an impressive decrease of -1.23% at 1-year post enrollment.48 The initial TExT-MED patient focused curriculum showed a 0.8% decrease among Spanish-speaking emergency department patients at 6 months after enrollment.9 The critical link in addressing language and cultural barriers to adequate continuity care for diabetes management may not be fully addressed in individual level interventions such as SMS text-message; integration into medical homes may be required to improve patient outcomes in linguistically underserved communities."

21-ii) Discuss if there were elements in the RCT that would be different in a routine application setting

Discuss if there were elements in the RCT that would be different in a routine application setting (e.g., prompts/reminders, more human involvement, training sessions or other co-interventions) and what impact the omission of these elements could have on use, adoption, or outcomes if the intervention is applied outside of a RCT setting.

	1	2	3	4	5	
subitem not at all important	○	○	○	○	○	essential

Does your paper address subitem 21-ii?

Copy and paste relevant sections from the manuscript (include quotes in quotation marks "like this" to indicate direct quotes from your manuscript), or elaborate on this item by providing additional information not in the ms, or briefly explain why the item is not applicable/relevant for your study

"TExT-MED for patients with or without a social support component has the potential to improve the long-term health outcomes of these vulnerable patients and has high potential to be translated to a system wide intervention, given the possibility of remote self-enrollment by patients."

OTHER INFORMATION

23) Registration number and name of trial registry

Does your paper address CONSORT subitem 23? *

Copy and paste relevant sections from the manuscript (include quotes in quotation marks "like this" to indicate direct quotes from your manuscript), or elaborate on this item by providing additional information not in the ms, or briefly explain why the item is not applicable/relevant for your study

"The full details of study design and procedures have been published previously,11 and are available at ClinicalTrials.gov (NCT03178773)."

24) Where the full trial protocol can be accessed, if available

Does your paper address CONSORT subitem 24? *

Cite a Multimedia Appendix, other reference, or copy and paste relevant sections from the manuscript (include quotes in quotation marks "like this" to indicate direct quotes from your manuscript), or elaborate on this item by providing additional information not in the ms, or briefly explain why the item is not applicable/relevant for your study

"The full details of study design and procedures have been published previously,11 and are available at ClinicalTrials.gov (NCT03178773)."

25) Sources of funding and other support (such as supply of drugs), role of funders

Does your paper address CONSORT subitem 25? *

Copy and paste relevant sections from the manuscript (include quotes in quotation marks "like this" to indicate direct quotes from your manuscript), or elaborate on this item by providing additional information not in the ms, or briefly explain why the item is not applicable/relevant for your study

"Funding statement
Funding for this trial was provided by NIDDK Award 5K23DK106538."

X27) Conflicts of Interest (not a CONSORT item)

X27-i) State the relation of the study team towards the system being evaluated

In addition to the usual declaration of interests (financial or otherwise), also state the relation of the study team towards the system being evaluated, i.e., state if the authors/evaluators are distinct from or identical with the developers/sponsors of the intervention.

	1	2	3	4	5	
subitem not at all important	○	○	○	○	○	essential

Does your paper address subitem X27-i?

Copy and paste relevant sections from the manuscript (include quotes in quotation marks "like this" to indicate direct quotes from your manuscript), or elaborate on this item by providing additional information not in the ms, or briefly explain why the item is not applicable/relevant for your study

"Conflicts of Interest
Drs. Arora, Menchine and Burner have received compensation as consultants and on advisory boards from Agile Health on SMS-based interventions for patient self-care."

About the CONSORT EHEALTH checklist

As a result of using this checklist, did you make changes in your manuscript? *

- ○ yes, major changes
- ○ yes, minor changes
- ◉ no

What were the most important changes you made as a result of using this checklist?

no changes were required, I am familiar with the checklist from prior publications and used it to guide my writing previously.

How much time did you spend on going through the checklist INCLUDING making changes in your manuscript *

60 min. this checklist format is too cumbersome. please move to a modern platform.

As a result of using this checklist, do you think your manuscript has improved? *

- ○ yes
- ○ no
- ◉ Other: used previously to write the manuscript

Would you like to become involved in the CONSORT EHEALTH group?

This would involve for example becoming involved in participating in a workshop and writing an "Explanation and Elaboration" document

- ○ yes
- ◉ no
- ○ Other: ____________________

Clear selection

Any other comments or questions on CONSORT EHEALTH

Your answer

STOP - Save this form as PDF before you click submit

To generate a record that you filled in this form, we recommend to generate a PDF of this page (on a Mac, simply select "print" and then select "print as PDF") before you submit it.

When you submit your (revised) paper to JMIR, please upload the PDF as supplementary file.

Don't worry if some text in the textboxes is cut off, as we still have the complete information in our database. Thank you!

Final step: Click submit !

Click submit so we have your answers in our database!

Submit

Clear form

Never submit passwords through Google Forms.

This content is neither created nor endorsed by Google. Report Abuse - Terms of Service - Privacy Policy
